# Comprehensive bioinformatics analysis of *Mycoplasma pneumoniae* genomes to investigate underlying population structure and type-specific determinants

**DOI:** 10.1371/journal.pone.0174701

**Published:** 2017-04-14

**Authors:** Maureen H. Diaz, Heta P. Desai, Shatavia S. Morrison, Alvaro J. Benitez, Bernard J. Wolff, Jason Caravas, Timothy D. Read, Deborah Dean, Jonas M. Winchell

**Affiliations:** 1 Respiratory Diseases Branch, Division of Bacterial Diseases, National Center for Immunization and Respiratory Diseases, Centers for Disease Control and Prevention, Atlanta, Georgia, United States of America; 2 Division of Infectious Diseases, Department of Medicine, Emory University School of Medicine, Atlanta, Georgia, United States of America; 3 Center for Immunobiology and Vaccine Research, University of California San Francisco Benioff Children’s Hospital Oakland Research Institute, Oakland, California, United States of America; 4 Joint Graduate Program in Bioengineering, University of California San Francisco and University of California Berkeley, Oakland, California, United States of America; Oklahoma State University, UNITED STATES

## Abstract

*Mycoplasma pneumoniae* is a significant cause of respiratory illness worldwide. Despite a minimal and highly conserved genome, genetic diversity within the species may impact disease. We performed whole genome sequencing (WGS) analysis of 107 *M*. *pneumoniae* isolates, including 67 newly sequenced using the Pacific BioSciences RS II and/or Illumina MiSeq sequencing platforms. Comparative genomic analysis of 107 genomes revealed >3,000 single nucleotide polymorphisms (SNPs) in total, including 520 type-specific SNPs. Population structure analysis supported the existence of six distinct subgroups, three within each type. We developed a predictive model to classify an isolate based on whole genome SNPs called against the reference genome into the identified subtypes, obviating the need for genome assembly. This study is the most comprehensive WGS analysis for *M*. *pneumoniae* to date, underscoring the power of combining complementary sequencing technologies to overcome difficult-to-sequence regions and highlighting potential differential genomic signatures in *M*. *pneumoniae*.

## Introduction

The human pathogen *Mycoplasma pneumoniae* is a leading cause of respiratory illnesses worldwide [1–3]. Infections can result in varied disease presentations in all age groups, ranging from mild to life-threatening, and may lead to extra-pulmonary manifestations, auto-immune phenomena, and exacerbations of asthma in children and adults [2–6].

At ~820 kb, *M*. *pneumoniae* has one of the smallest free-living bacterial genomes. While inter-strain comparisons have revealed a high degree of similarity within this species, some type-specific genomic variability has been described [7–11]. Traditionally, polymorphisms in genomic regions encoding the P1 adhesin molecule have been used to categorize *M*. *pneumoniae* into two distinct subgroups, type 1 and type 2 [12–14]; variants have also been described [7, 15–17]. Over the past decade, limited and incomplete genomic sequence data have been exploited to develop several strain typing systems, including various methods for P1 typing, multi-locus variable number tandem repeat analysis (MLVA), multi-locus sequence typing (MLST), and single nucleotide polymorphism (SNP) genotyping [17–24]. However, none of these methods have led to a conclusive classification schema predicated on the comprehensive analysis of a significant number of completed whole genome sequences.

More recently, comparative genomic studies of *M*. *pneumoniae* have provided a glimpse into key features of this organism and rapidly expanded the collection of publicly-available genomic, transcriptomic, and proteomic data [8, 11]. While these studies support a high degree of similarity among *M*. *pneumoniae* isolates, close examination indicated that sufficient genetic diversity exists to support further separation of strain types and suggested that this diversity may directly impact pathogenesis.

In the current study, we examined 107 *M*. *pneumoniae* genomes, of which 67 were newly sequenced using a combination of long and short read sequencing data. We identified type-specific SNPs and genomic regions that contribute to an underlying population structure consisting of six distinct subtypes and utilized a supervised machine-learning technique to rapidly classify *M*. *pneumoniae* genomes into these types.

## Materials and methods

### *M*. *pneumoniae* isolates

Sixty-seven *M*. *pneumoniae* isolates were selected from the historical strain collection stored in the Pneumonia Response and Surveillance Laboratory, Respiratory Diseases Branch, Centers for Disease Control and Prevention (CDC), Atlanta, GA, USA (S1 Table). Isolates were selected to represent various clinical manifestations, broad temporal and geographical distributions, and diverse strain characteristics as determined using existing typing methods, including P1 typing by PCR with HRM, MLVA, and macrolide susceptibility genotyping [20, 25–27]. Reference strains M129 (type 1) and FH (type 2) obtained from American Tissue Culture Collection (ATCC) were also resequenced in this study; two isolates of FH procured in different years were included.

*M*. *pneumoniae* isolates were grown in SP4 media (Thermo Fisher Scientific, USA) as previously described [28]. Two mL cultures were used to seed large volume (30 mL) cultures and incubated for 10 days to obtain sufficient material for preparation of genomic gDNA libraries. Genomic DNA was extracted from bacterial cultures using the MasterPure Complete DNA and RNA Purification Kit (Epicentre, Madison, WI) according to manufacturer’s protocol.

### Publicly-available *M*. *pneumoniae* genomes

Forty *M*. *pneumoniae* genomes were available from the National Center for Biotechnology and Information (NCBI) data repository and downloaded on 09/06/2015 (S1 Table). The genomes used as references in the bioinformatics analysis were: *M*. *pneumoniae* M129 (Accession: NC_000912.1), *M*. *pneumoniae* M129-B7 (Accession: NC_020076.2), *M*. *pneumoniae* FH (Accession: NZ_CP010546.1), and *M*. *pneumoniae* 309 (Accession: NC_016807.1) (S2 Table).

### Illumina whole genome sequencing and assembly

The NEBNext Ultra DNA library prep kit for Illumina (New England Biolabs, Ipswich, MA) was used to prepare gDNA libraries for each isolate according to the manufacturer’s protocol. Whole genome sequencing was performed on the 67 *M*. *pneumoniae* isolates using the Illumina MiSeq desktop sequencer (Illumina, San Diego, CA) with Illumina MiSeq version 2.0.

FastQC version 0.10.1 [29] was used to evaluate Illumina sequencing read quality. Sequence read data cleansing was performed with Cutadapt v1.5 [30]. Sequencing reads were removed from the data set if they met one of the following criteria: (i) had low quality (< 25) sequence bases; (ii) trimmed adapter sequences; (iii) had an error rate above 0.03; or (iv) were < 75 base pair in length. *De novo* assembly was performed with VelvetOptimiser v2.2.5 [31] and Velvet v1.2.10 [32]. The iterative assembly process to identify the optimal kmer value is described in detail in S1 Text.

### Pacific Biosciences whole genome sequencing and assembly

A subset (n = 25) of the 67 isolates representing various outbreaks, macrolide resistance genotypes, geographic origins, and patient outcomes/disease characteristics were selected for long-read sequencing using the Pacific Biosciences (PacBio) RS II (Pacific Biosciences, Menlo Park, CA) (S1 Table). One or two SMRT cells were used for each isolate with a movie time ranging from 120–240 minutes. The sequences were assembled with SMRT Analysis v2.2 Hierarchical Genome Assembly Process version 2 (HGAP 2) or 3 (HGAP 3) protocol [33]. The Illumina sequencing data associated with each isolate was aligned for nucleotide accuracy comparison (S1 Text). The final sequences were re-oriented to start with the DNA polymerase III subunit beta gene (*dnaN*, locus tag–MPN001). The PacBio consensus sequences were used in place of Illumina assemblies for those 25 isolates.

### Data availability

All sequencing data and assembled genomic sequences generated in this study were deposited in NCBI Genome and Short Read Archive (SRA) databases and are available under BioProject ID PRJNA328832 (S2 Table).

### Genomic structure variation

BLAST Ring Image Generator (BRIG) was used to visualize comparisons of reference genomes and complete closed genomes [34]. Mauve v2.4.0 [35] was used to identify differences between the genomic structures of type 1 and type 2 isolates. Seven permutations of alignments were performed to observe structural arrangements to ensure that type-specific genome structures were captured with draft genome content as described in S3 Table. Alignment using the first dataset was performed to observe the genomic structure between closed genomes only, and alignments using second and third datasets were used to confirm consistency of regions across isolates. The remaining four datasets were constructed with draft genomes. Closed genomes from Xiao *et*.*al* [11], assembled from PacBio in this study, and NCBI references *M*. *pneumoniae* M129-B7 (Accession: NC_020076.2) and *M*. *pneumoniae* FH (Accession: NZ_CP010546.1) were aligned with Mauve v2.4.0 [36]. Backbone files derived from progressiveMauve [35] were analyzed to identify regions that were present in all type 1 genomes and absent in all type 2 genomes and the reciprocal. Each of these regions identified with backbone file were compared using BLAST against a sequence database constructed with whole genome sequences in order to verify that these sequences were type-specific. The criterion to identify these regions were as follows: >75% identity and >75% query coverage in any inter-type genome (compared to the reference type). We further used BLAST results to identify any hits that were identified to be only partial query hit (less than 40% coverage) compared to the region of interest. Any genes present in type-specific genomic regions that encoded hypothetical proteins were analyzed using Interproscan for prediction of motifs and protein function [37].

### Gene prediction and phylogenetic analysis

Prodigal v2.60 [38], an *ab initio* gene finder, was used to predict protein coding sequences. Since *M*. *pneumoniae* has a non-traditional translation code [39], parameter “–g” was changed to 4 to allow for translation suited for *Mycoplasma* species. Also, protein predictions were restricted to closed ends by using parameter “-c”, which does not allow for partial gene predictions on the ends of contig sequences. To identify recombinant sites in shared genome, an alignment (n = 107) was used with Gubbins v.1.4.1 with default settings [40]. The whole genome alignment was not used because the dataset included both broken contigs and closed genomes and could result in over-representation of missing intergenic regions or create large misaligned regions.

PanOCT v3.23 [41], a sequence clustering tool designed for closely related species or strains, was used to determine the orthologous relationships between the isolates. A distinctive feature of PanOCT is the ability to differentiate paralog sequences with gene synteny [41]. Orthologous clusters were stringently defined as all sequences in a cluster having shared sequence identity and coverage ≥ 75% (S1 Text). The core genome was extracted from the pan-genome dataset to construct a phylogenetic tree. ClustalOmega v1.2 [42] was used to perform multiple sequence alignments on each set of genes in a cluster defined by PanOCT in order to avoid potential gene rearrangements in the sequence concatenation step. All individual aligned protein sequences were concatenated together to construct the core sequences for phylogenetic analysis using the method described by Hasan *et al*. [43]. A ML phylogenetic tree was constructed using RAxML v7.3.0 [44] with the following parameters: JTT matrix-model with the г model for rate heterogeneity, GAMMA model for four discrete rate categories, and 1000 bootstrapping replicates. Figtree [45] and the MEGA6 application [46] were used to visualize the tree. The core genome determination and subsequent phylogenetic analysis was performed on two datasets: (i) all available genomes regardless of origin and method of sequencing (n = 107) and (ii) closed genomes only (n = 34) (S1 Table).

### single nucleotide polymorphism (SNP) analysis

SNPs were identified using Bowtie2 and Freebayes for the isolates that had Illumina data only. First, Illumina sequence reads from each isolate were aligned to the appropriate P1 type reference sequence using Bowtie2 with default parameters [47]. Secondly, Freebayes was used to identify SNPs based on allele coverage (> 95%) and nucleotide quality on the Phred score (>25%). Positive variant calls with likelihood score of zero were classified as false positive variants and were removed from the remainder of the analysis. Finally, all corresponding SNPs were merged based on the type reference genome position. For the 25 isolates sequenced using PacBio RSII and the isolates included in Xiao *et al*. [11], NUCmer and the show-snps utility from the MUMmer version 3.0 application were used to identify SNPs. For show-snps a–C parameter was chosen to remove the variants with ambiguous mapping as well as–I to exclude indel calls.

### Synonymous and non-synonymous mutation rate analysis

Gene clusters were codon aligned using Multiple Alignment of Coding SEquences (MACSE) version 1.01b [48]. After alignment, a custom Perl script was used to strip in-frame stop codons and the alignments were analyzed with Phylogenetic Analysis by Maximum Likelihood package version 4.5 [49]. The codeml program was run on each single gene cluster as well as the concatenated 464 gene core genome set using the tree topology generated above and a free ratio model (settings model = 1, NSsites = 0, icode = 3, fix_omega = 0, omega = 0.4, fix_alpha = 1, alpha = 0, ncatG = 3, cleandata = 1, fix_blength = 1, method = 1). The result files were analyzed with a custom Perl script to locate branches with sufficient information to generate a meaningful estimate of ω (defined here as S * *d*_S_ ≥ 2).

### Population structure analysis

The hierarchical Bayesian Analysis of Population Structure (hierBAPS) utility [50] was used to analyze the population structure of all genomes (n = 107), closed genomes (n = 34), type 1 genomes (n = 56), and type 2 closed genomes (n = 20). Independent core nucleotide genome alignments were generated for type 1 and type 2 isolates based on type determined by established laboratory methods as previously described for the core genome. The maximum cluster number was set to 15, and maximum hierarchical runs were set to 10–20.

### Genome classification with Random Forest

The whole genome SNP matrix and the classifications identified in the population structure analysis were used as input for the Random Forest (RF) analysis to construct a composite matrix (S4 and S5 Tables) in which population subgroups identified in the hierBAPS analysis were in columns and allele variants for each site were in rows. The input matrix was reduced to 659 lists each containing 1–520 variant positions within the genome that shared the same SNP profile across 106 genomes (S5 Table). Variant calls were converted to a binary representation for presence (1)–absence (0) for each location (S4 Table).

Four-fold cross-validation method was used to build a classification model; the input matrix was randomly divided into three-fourths for training and one-fourth for testing the resulting model. The dataset included >3000 variant features and approximately 70 genomes depending on randomly divided three fourths dataset in each fold. The RF training model consisted of randomized feature selection with 1000 trees using RandomForest v4.6–12 [51] with R version 3.2 [52]. Ten iterations of four-fold cross validation were performed with randomly assigned testing and training datasets at each fold. At each iteration, decrease in Gini index was used to identify features of highest importance.

## Results

### Genome assembly and characteristics

Assembly characteristics and genome characterization features for each genome included in this study are summarized in S1 Table. As expected, Pacific Biosciences (PacBio) Single-Molecule Real-Time (SMRT) sequencing resulted in longer read lengths and fewer contigs but lower average genome depth coverage compared to Illumina sequence data. Complete closed genomic sequences were generated for 17 of 25 (68%) isolates sequenced with the PacBio platform; the remaining eight consisted of ≤ 6 contigs. Nucleotide accuracy of PacBio sequences was > 99.85% as determined by alignment with Illumina reads. On average, 759 genes were predicted in the resulting genome sequences, which was comparable to the results of available *M*. *pneumoniae* reference genomes [11, 53]. Using Gubbins, 19 regions were identified as potential recombination sites (all <300 bp), yet the phylogenetic tree remained unchanged after masking these regions (data not shown) and thus was not considered supportive evidence of recombination driving the evolution of *M*. *pneumoniae*.

We initially compared available genome sequences of the prototypical reference strains of *M*. *pneumoniae*, M129 (type 1) and FH (type 2), to those re-sequenced in the current study in terms of both genomic content and SNPs (S1 Fig). The original FH reference genome (NC_017504.1) lacked a 6 kb region shown to be present among newer constructs of the FH genome and all type 2 isolates [11], including those in the current study (n = 51). Thus, we used the sequence reported by Xiao *et al*. [11] (NZ_CP010546.1) as the reference genome for type 2 isolates for the remaining analysis. The type 1 strain M129 was >99.99% identical in nucleotide sequence to recent reference sequence (NC_020076.2) that was used as type 1 reference genome in the current study (S6 Table).

### Type-specific genomic content

Initial genome-wide alignments of type 1 and 2 reference genomes revealed six segments ranging in size from 13 bp to over 5400 bp in length that were unique to one type. The largest segment was previously identified by Xiao *et al*. [11] as a type 1-specific insertion sequence consisting of genes encoding hypothetical proteins and a single tRNA gene. Three small indels (13–15 bp) previously identified as unique to either type 1 or type 2 isolates [54] were confirmed in all newly sequenced genomes (n = 67). To confirm the apparent absence of these segments was not due to lack of Illumina sequencing reads, we performed multiple sequence alignments of only closed genomes (n = 34) (Table 1). We identified three larger regions present in all type 1 and absent in all type 2 genomes and one region present in all type 2 and absent from all type 1 genomes. However, BLAST analysis showed that these regions contained partial repetitive genomic content that were not unique to that type; hence, we examined these regions more closely to identify the sections of unique genomic content of at least 100 bp (Table 1). Portions of the single large type 2-specific region were also present in type 1 genomes, thus this segment was divided into three smaller regions that were truly unique to type 2 isolates. These regions were examined further to identify gene content and putative or known function of affected genes, which included lipoproteins, hypothetical proteins, and pseudogenes (Table 1).

**Table 1:** Type-specific genomic regions and gene content.

**Query reference block**	**Start position**^1^	**End position**^1^	**Size (bp)**	**Genes in region**	**Known or predicted function**
**Type 1-specific**					
NC_020076.2_177739–178611	177937	178611	674	1. C985_0138^2^ 2. C985_RS00830^2^	1. DUF16 Superfamily Protein 2. DUF16 Superfamily Protein
NC_020076.2_558159–561575	559124	559535	411	C985_RS02615^2^	pseudogene
NC_020076.2_558159–561575	561433	561575	142	C985_RS02615^2^	pseudogene
**Type 2-specific**					
CP010546.1_703624–709505	704334	705013	679	F539_03315^2^	Putative Peptidase- DUF31 Superfamily
CP010546.1_703624–709505	705827	706542	715	F539_03320^2^	Lipoprotein–DUF31 Superfamily
CP010546.1_703624–709505	707053	709505	2452	1. F539_03325 2. F539_03330^2^	1. Hypothetical Protein (Putative Peptidase DUF31; Peptidase S1, PA Clan–Trypsin-like Serine Proteases) 2. Putative Lipoprotein-DUF31 Superfamily

^1^ Refers to start and end of unique region within query reference block

^2^ Partial gene

### Phylogenetic analysis of core genomic content

**Fig 1:**
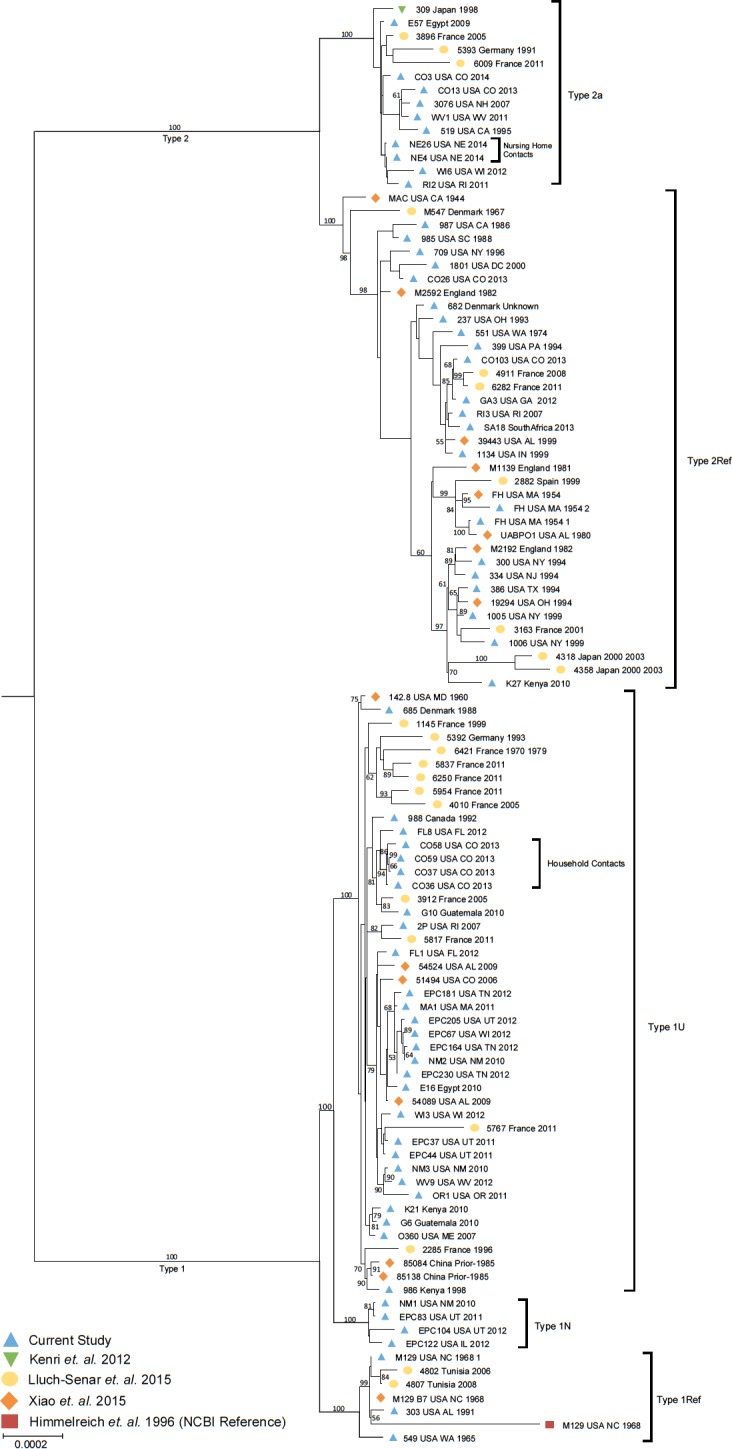
Phylogenetic tree of all *M*. *pneumoniae* isolates in the current study (n = 107). Maximum likelihood phylogenetic tree of 107 *M*. *pneumoniae* isolates based on core protein sequences (n = 464) identified through orthologous clustering. Bootstrapping values over 50 are represented on the tree.

The *M*. *pneumoniae* core genome dropped from 595 proteins to 464 upon inclusion of assembled contigs from Lluch-Senar *et*. *al* [8] (S2 Fig). The core used for the primary analysis of all 107 isolates included 464 proteins compared to 642 proteins for analysis of closed genomes only (S2 Fig). The lower number for the non-closed genomes was most likely due to mis-assembly, particularly at repetitive regions. The 464 core protein sequences were used to construct a maximum likelihood (ML) phylogenetic tree to represent the genomic relatedness of isolates included in this study (Fig 1). The phylogenetic analysis revealed a strong separation of isolates into clades corresponding to the known P1 type classifications based on previous laboratory testing methods. Two branches were evident within the type 1 clade; one included the majority of type 1 isolates (n = 49, 87.5%), which we designated as type 1U (**U**biquitous), and the other consisted of three genome representations of type 1 reference strain M129, along with four isolates, 303 (AL, USA), 549 (WA, USA), 4802 and 4807 (Tunisia), which we designated type 1Ref (Fig 1). The two isolates from Tunisia were previously reported as a distinct subtype designated 1d [8]. Within the larger type 1 branch, four isolates from the current study (EPC83, EPC104, EPC122, and NM1) formed a distinct subgroup designated 1N (**N**ew) (Fig 1). These four isolates originated from clinical cases occurring in the United States between 2010 and 2012. Although initially identified as MLVA type 4570 or 5570, WGS analysis revealed the presence of three tandem repeats at *mpn16*, the final VNTR included in the MLVA scheme, instead of zero (S3 Fig). Review of the original MLVA electropherograms for these isolates revealed the appropriate size peak corresponding to three repeats at this position, which was masked in the initial analysis by a peak of nearly identical size corresponding to repeats at a separate locus. The three repeats at this locus are a distinguishing feature from all other isolates in this study.

Further phylogenetic separation of type 2 genomes within the large clade was also observed. Two main clusters were identified with one branch including all versions of the type 2 reference strain FH along with 27 isolates, all previously identified as type 2 through laboratory testing methods (designated here as type 2Ref), and the other containing the type 2 reference strain 309, which previously has been proposed as a new variant type, 2a [7] and 13 other isolates (Fig 1). Three isolates (3896, 5393, and 6009) from Germany and France previously designated as type 2 variants designated 2d by Lluch-Senar *et al*. [8] were also present in the clade with 309 and were identified as type 2a for the purpose of this study. A number of isolates reported as a variant of type 2 by high-resolution melt analysis of a 1900 bp amplicon within P1 were also included in this analysis [17]. Interestingly, these did not form a distinct separate cluster in the tree based on 464 core proteins; however, clear separation of these isolates was observed when the phylogenetic tree was generated using a kmer based SNP method (S4 Fig). This group consisted of isolates from the United States (n = 5), South Africa (n = 1), and France (n = 2), all collected since 2000. The two isolates from France (4911 and 6282) were previously reported as a separate subgroup designated 2c as determined by SNP/indel analysis [8]. Consistent with previous nomenclature used in our laboratory, this branch was designated as type 2 **v**ariant (2v).

**Fig 2:**
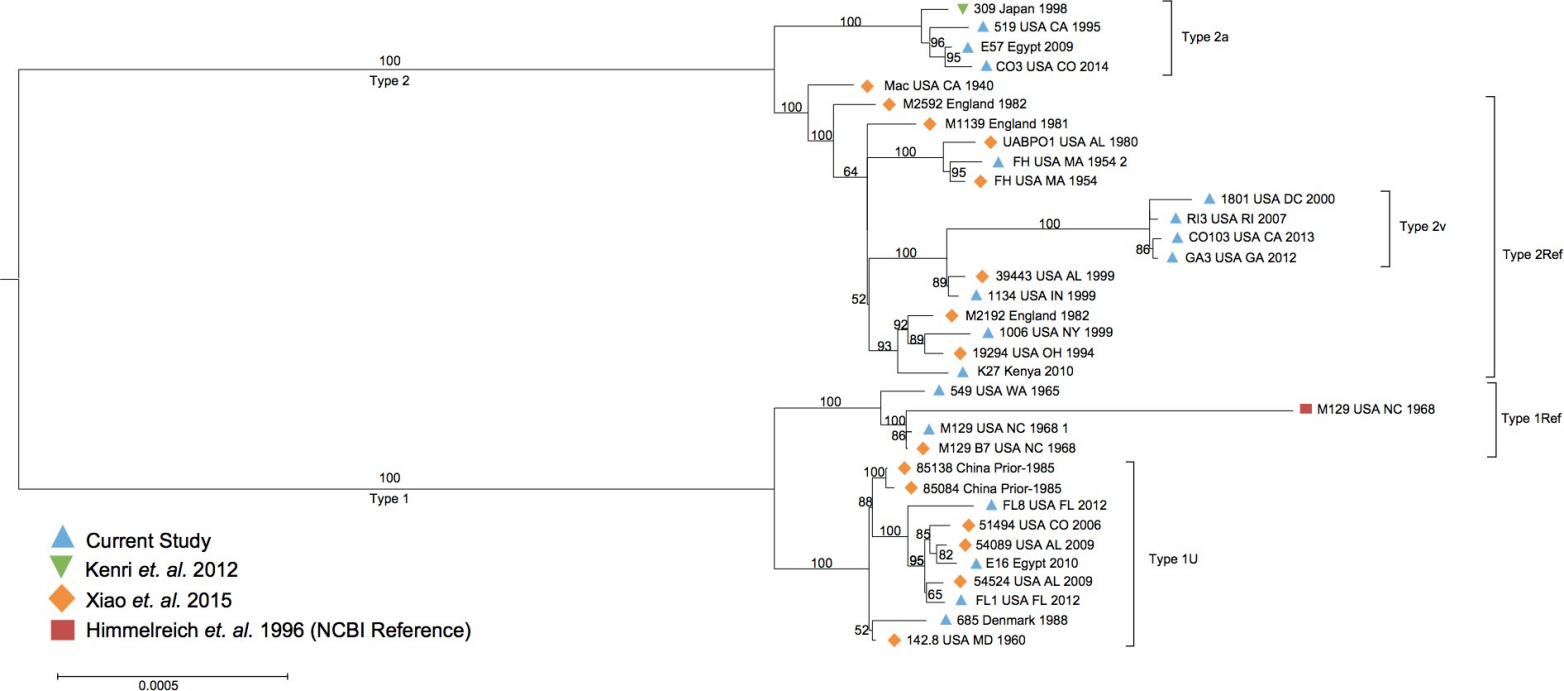
Phylogenetic tree of closed *M*. *pneumoniae* genomes in the current study (n = 34). Maximum likelihood phylogenetic tree of 34 *M*. *pneumoniae* isolates based on core protein sequences (n = 642) identified through orthologous clustering. Bootstrapping values over 50 are represented on the tree.

The tree based on 464 core proteins (using closed and non-closed genomes) was found not to separate one of the subgroups (2v) from other type 2 genomes. Hence, phylogenetic analysis was performed using only the 34 closed genomes (S1 Table) based on the core proteins determined from this subset of genomes (n = 642, 80% of the total genes). This analysis also revealed the two expected clades consisting of P1 types 1 and 2 (Fig 2). The clade consisting of type 2v isolates from our laboratory was evident in the tree generated from closed genomes, similar to the kmer based SNP analysis (S4 Fig). The distinction of this type may be attributed to the approximately 178 additional core proteins included in the closed genome analysis (S7 Table), which represents approximately 30% more sequence data compared to the core protein set used for the primary phylogenetic analysis.

Further distinction of epidemiologically-related isolates was also observed. For example, the dominant type 1 subgroup (1U) contained the vast majority of isolates obtained from surveillance and outbreak investigations between 2010 and 2013, a period during which increased *M*. *pneumoniae* activity was observed worldwide [55–57]. The four isolates noted as type 1N (Fig 1) were an exception to this finding, having all been collected during this same period. Notably, isolates related by close association of patients, such as household contacts with *M*. *pneumoniae* infection (CO36/37 with CO58 and CO59; CO3 with CO13) [58] or cases from an outbreak in a long-term care facility (NE4 and NE26) [59] were found to be closely related.

Inherent limitations of short-read sequencing may impact resolution of repetitive sequences, several of which are known to exist in *M*. *pneumoniae*, including repetitive elements within P1. To explore the added value of whole genome sequencing data, we performed a phylogenetic analysis on a portion of the P1-encoding sequence extracted from closed genomes (S5 Fig). We observed a clear separation of the same three variant subgroups within the type 2 clade identified in the primary phylogenetic analysis using WGS data; however, no clear separation of subgroups was observed for type 1 isolates (S5 Fig).

### SNP variant analysis

**Fig 3:**
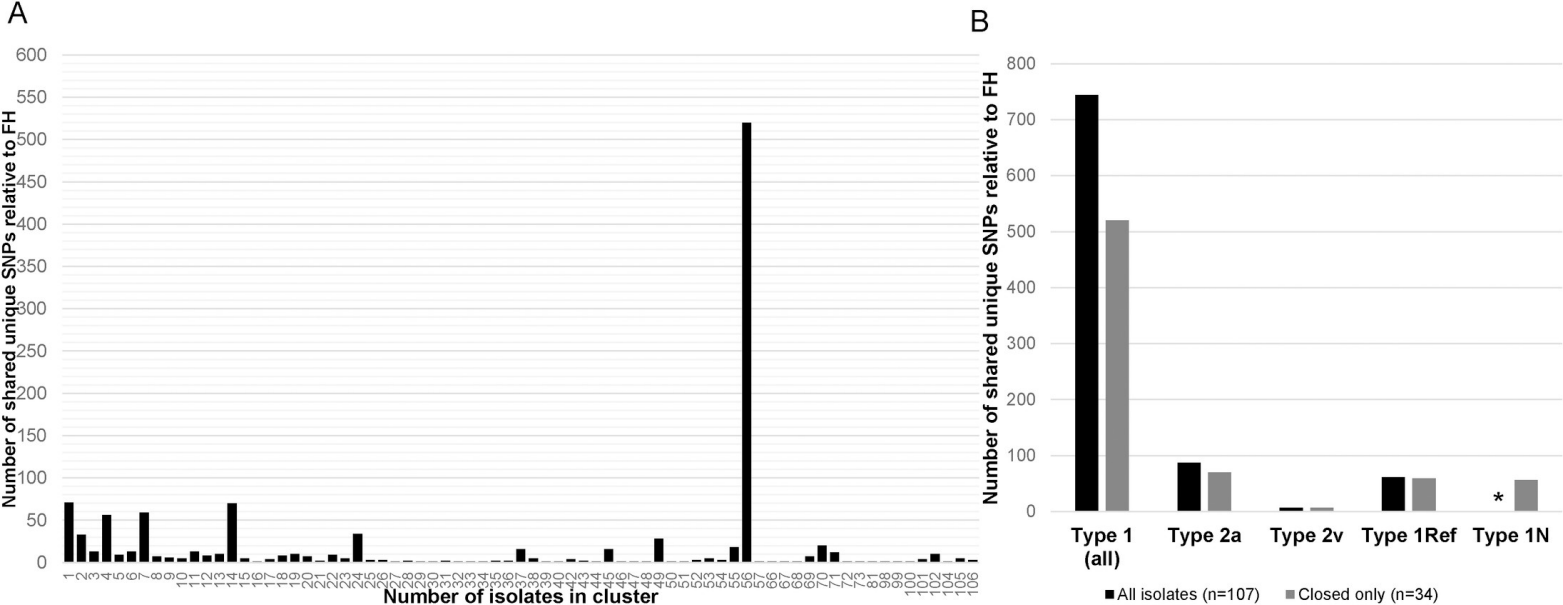
Clusters of *M*. *pneumoniae* isolates sharing unique SNPs. (A) Number of shared unique SNPs in isolate clusters ranging from 1–106 isolates relative to reference genome FH. Only the group of isolates sharing the largest number of SNPs is shown. (B) Number of shared SNPs in each subtype relative to type 2 reference FH identified among all genomes (black bars) or closed genomes only (grey bars). *No closed genomes were available for Type 1N.

We identified a total of 3,206 SNPs present in at least one isolate. Intra-type examination of SNPs revealed 889 SNPs present in at least one type 1 isolate relative to the type 1 reference and 942 SNPs in one or more type 2 isolates relative to the type 2 reference genome. However these SNPs were not consistent amongst all isolates within the type designations. Comparing all 107 isolates, 520 SNPs were identified as consensus alleles in all isolates within one type group as compared to all isolates of the other type (Fig 3, S8 Table). Of these, 470 (90.4%) were located in coding regions. These 520 SNPs led to clear separation of isolates corresponding to known P1 type based on laboratory methods. Interestingly, the primary gene encoding P1 (mpn141) was not included in the core genome identified using all 107 isolates, although it was present in the core based on closed genomes only. Thus, the separation observed in our analysis must result from genomic variation outside of this gene. Other subgroups identified in the phylogenetic analysis varied from the FH reference genome by 70 (type 2a), 59 (type 1Ref), 56 (type 1N), or 7 (type 2v) SNPs that are unique to that subtype (Fig 3B). When comparing closed genomes only, the number of SNPs between the large type 1 and 2 groups was 744, presumably due to the inclusion of a larger number of core proteins; subtype-specific SNPs were similar using either core dataset (Fig 3B).

SNPs associated with macrolide resistance were identified only in strains previously reported to harbor these mutations or have a laboratory-determined resistant phenotype. Verification of these known SNPs, including A2063G, A2064G, and C2611G mutations in the 23S rRNA gene, increased our overall confidence in variant calls.

We confirmed the presence of a type-specific non-synonymous SNP in the CARDS toxin gene (T1112G) resulting in the I371S substitution that was previously reported [8, 11] (S6 Fig); this SNP was consistently found in all 67 newly sequenced isolates. In addition, one other non-synonymous SNP (C217T) was identified in the CARDS toxin gene of four isolates from the current study (CO36, 37, 58, and 59) (S6 Fig). This SNP results in a P73S substitution that is located at the beginning of the S4 beta-sheet region in the D1 mART domain. These four isolates include two recovered from upper respiratory tract specimens collected sequentially from a patient with *M*. *pneumoniae* infection during an epidemic in Colorado in 2013 [60] (CO36 and 37) as well as two isolates recovered from household contacts of this patient (CO58 and 59) [58]. Interestingly, several other isolates obtained from patients during the same outbreak period did not harbor this same mutation. In addition, ten other SNPs outside of the CARDS toxin gene were shared by these four isolates relative to all other isolates in the study (data not shown).

### Synonymous and non-synonymous mutation rate analysis

Our preliminary analysis of 1408 SNPs located in the 464 gene core genome showed a bias towards non-synonymous substitutions (61.93%), but this was expected based on the high degree of relatedness of *M*. *pneumoniae* genomes [61]. To identify whether these non-synonymous substitutions were accumulating in a manner suggestive of adaptive evolution on some branches, we analyzed gene clusters for evidence of selection (S1 Text). We identified two genes, DNA Ligase (ALA35866.1) and DNA Polymerase III subunit alpha (ALA35550.1), with a higher number of non-synonymous substitutions, potentially suggesting that purifying selection may be relaxed on these genes or that positive selection may be acting upon a subset of residues in these genes.

### Population structure analysis and predictive model

Based on the high-degree of shared genomic content between the various types of *M*. *pneumoniae* genomes, we evaluated the genetic structure to elucidate novel features that could be used to refine the *M*. *pneumoniae* classification schema. Consistent with divergent lineages of type 1 and 2 isolates documented previously [8, 11], the phylogenetic analysis revealed two population substructures that followed the primary P1 type classifications (Fig 1, Fig 2, S4 Fig). Initial hierarchical Bayesian Analysis of Population Structure (hierBAPS) using core sequences from all 107 isolates revealed 3 subgroups corresponding to type 1, type 2, and type 2a (S7 Fig). Considering the larger core consisting of 642 genes, two subtypes corresponding to the larger type 1 and type 2 groups were identified (S7 Fig).

**Fig 4:**
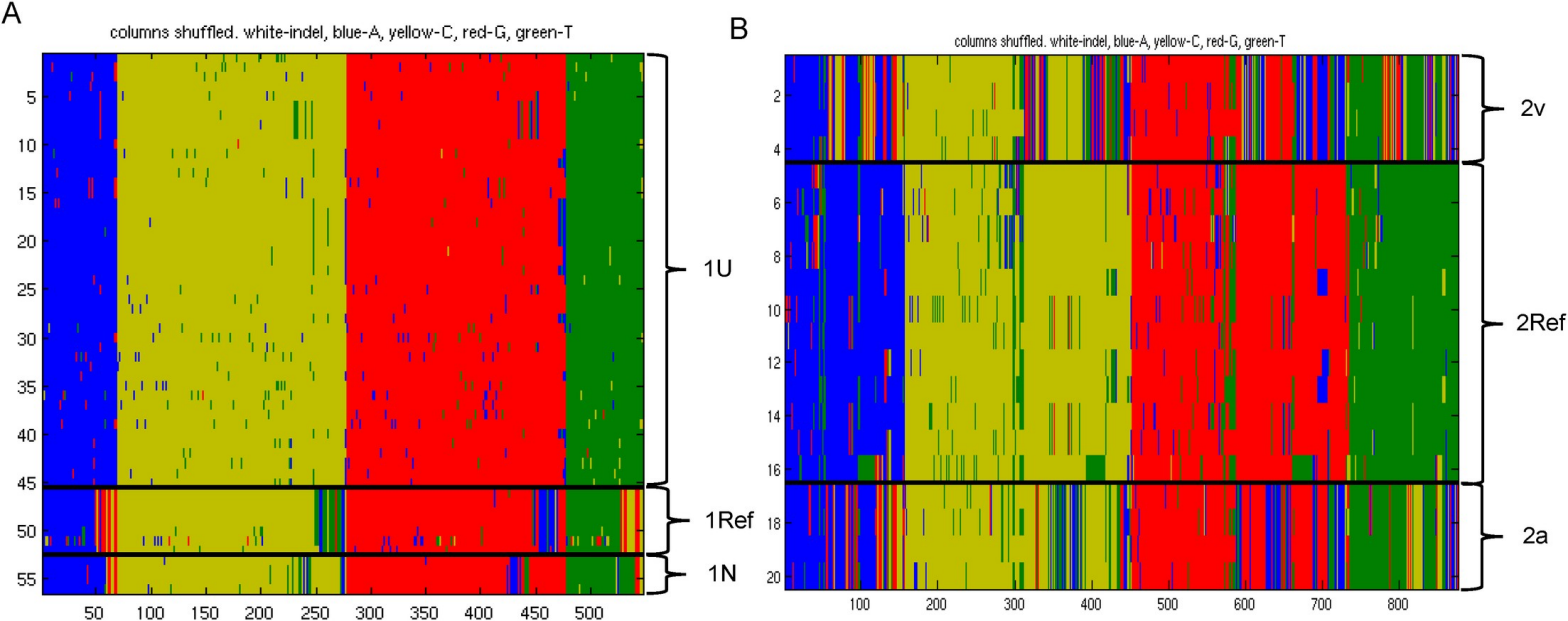
Hierarchical Bayesian Analysis of Population Structure (hierBAPS) of type 1 and type 2 groups separately. Three subpopulations were identified in (A) Type 1 and (B) Type 2 genome groups. Green, T; blue, A; red, G; yellow, C.

In order to reveal hidden substructures masked by the dominant population structure, we performed hierBAPS on type 1 and 2 datasets separately (Fig 4). For type 2, core nucleotide sequences (n = 642) obtained from closed genomes (n = 34) were used whereas, for type 1, the core genes (n = 464) identified in all isolates (n = 107) were used as none of the type 1N subgroup genomes were completely closed by WGS and thus would not have been represented in the dataset. Using hierBAPS we identified a total of six subpopulation structures, including three groups within type 1 (Fig 4A) and three within type 2 (Fig 4B) corresponding to the groups identified in the phylogenetic analysis. The groups within type 1 each contained the same isolates as the subtypes described in the phylogenetic analysis: type 1U (containing the majority of type 1 isolates), type 1Ref (containing genome representations of M129), and type 1N (containing newly emerging isolates with 3 repeats in the MLVA locus mpn16) (Fig 4A). Similarly, the three groups within type 2 corresponded to the previously identified subtypes, and thus were designated as type 2Ref (containing genome representations of FH), type 2a (containing isolate 309 originally designated as variant type 2a), and type 2v (containing isolates designated by our laboratory as variants of type 2) (Fig 4B).

**Fig 5:**
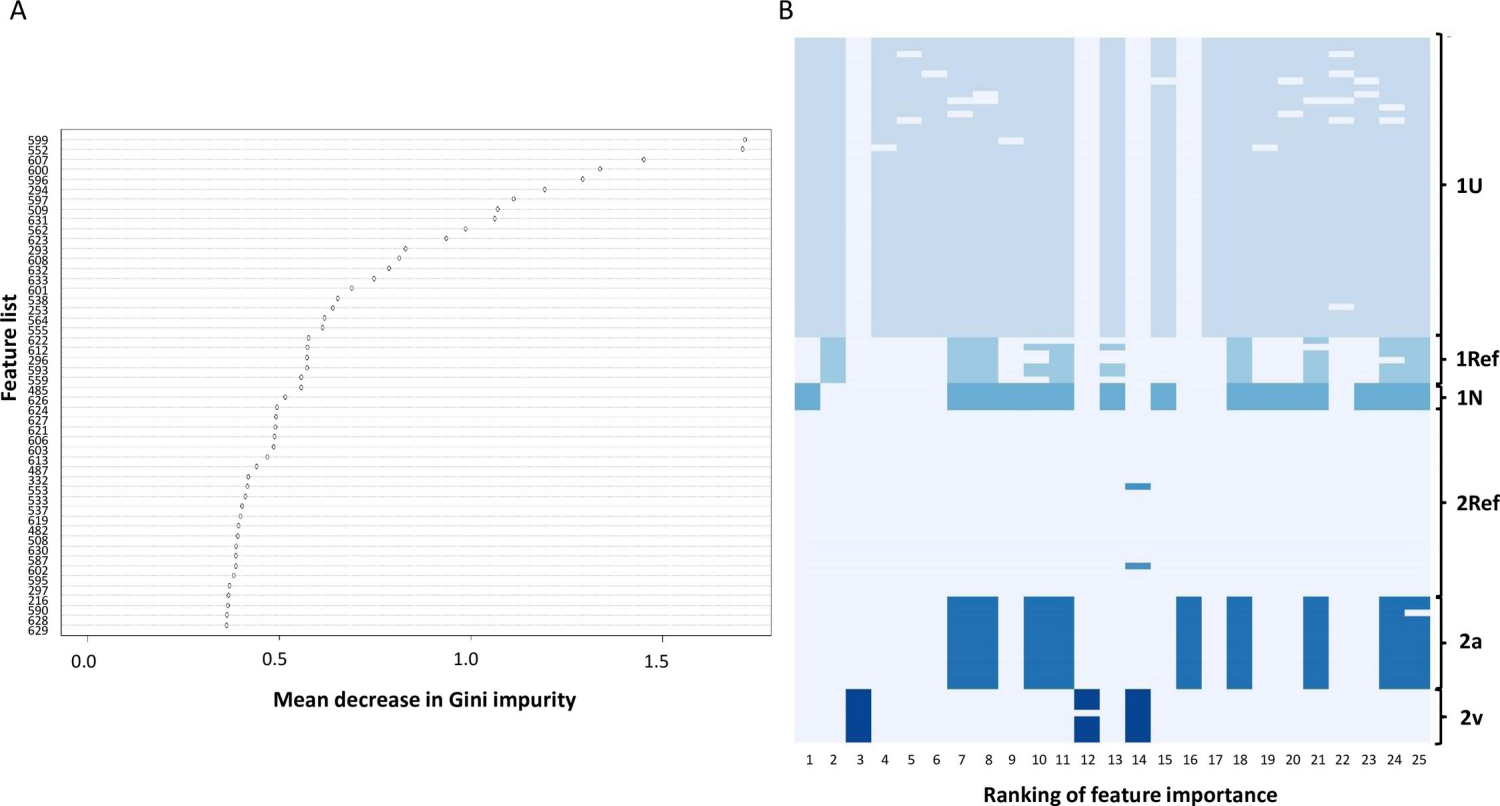
High importance features for classification of *M*. *pneumoniae* subtypes based on Random Forest model. (A) Representative plot of mean decrease in Gini value for top 50 feature lists. Each list consisted of ≥ 1 variant position. (B) Heatmap of presence/absence of the 25 features of highest relative importance for separation of six subtypes resulting from all iterations of the model (n = 40). Other variant sites also contributed to the final model.

In order to identify the SNPs that were used to classify isolates in the hierBAPS utility, we constructed a predictive classification model for whole genome SNP output using the Random Forest supervised machine learning algorithm with the subtypes as an isolate classifier and lists containing at ≥1 SNP as features (S4 and S5 Tables). We performed ten iterations of four-fold cross validation to train and test the model using a different seed for each iteration. The accuracy of the models ranged from 92.5% to 100%. In the represented model, only three misclassifications were observed (training, n = 2; testing, n = 1); each involved the subgroups containing the smallest number of representative isolates, including type 1N and type 2v. Of the 659 lists that contributed to the model (S5 Table), the relative importance of each feature list for classifying the data was determined as indicated by the mean decrease in Gini index value (Fig 5A, S9 Table). The presence/absence of features with the greatest contribution to differentiating the subtype classifications in all iterations of the model were identified (Fig 5B).

## Discussion

Recent WGS analyses have supported the long-standing concept of a highly conserved *M*. *pneumoniae* genome signified by its minimal size and based on previous evaluations using limited sequence data [8, 11, 62–64]. Xiao *et al*. reported >99% similarity among *M*. *pneumoniae* strains and noted the lack of evidence for horizontal gene transfer (HGT) with other species [11]. We also observed an overall high degree of similarity among our 107 strain collection, lack of HGT, and a clear separation of isolates into two clades corresponding to P1 types 1 and 2. Consistent with previous reports, we also identified specific genomic regions unique to either type 1 or type 2 isolates [10, 11, 65]. Based on careful review, we confirmed truly type-specific content, excluding portions of these regions that may be present elsewhere in the genomes of the other type. Thus, the size of type-specific regions may differ from previous reports [8, 10]. This close evaluation allowed us to develop a novel assay based on short unique sequences for typing *M*. *pneumoniae* [54]. Additional type-specific genomic content identified or confirmed in this study may also be used for development of improved molecular diagnostics to further differentiate *M*. *pneumoniae* isolates.

Historically, sequence variation within P1 has proven useful for rapid detection but limited molecular typing of *M*. *pneumoniae*. However, this study and others indicated that further differences identified through WGS provide a higher resolution and discriminatory power for differentiation of *M*. *pneumoniae* strains [8, 18, 20, 24]. The 520 conserved SNPs that differed between type 1 and 2 isolates account for only ~0.05% of the genome. Analysis of synonymous and non-synonymous mutations identified two genes, DNA ligase and DNA polymerase III subunit alpha, as possibly being under positive selection. However, the underlying pressures impacting changes in this essential proteins are not clear. Interestingly, one non-synonymous SNP was previously identified within the CARDS toxin gene of type 1 and 2 isolates [8, 11], and expression or stability of this toxin has been proposed to differ between the two types [8]. We identified another non-synonymous mutation in the toxin gene that was unique to several isolates from household contacts with *M*. *pneumoniae* infection. The type-specific differences in both genomic content and SNP/indel level variation that correlate with strain divergence or emerge in a small group of isolates with potential to expand to a larger population, warrant further examination.

Phylogenetic and population structure analyses supported a sufficient level of diversity to further classify strains within the main subgroups of type 1 and 2, including three distinct subgroups. In a previous study, type-specific genomic variation was found to be enriched in specific areas of the genome, including P1 and the related gene ORF6, which together form the P1 adhesion molecule [11]. Interestingly, the core genome generated using an orthologous clustering method did not include P1, potentially due to inaccurate or incomplete genome assemblies resulting from repetitive elements within this region. Hence, separation of strains into types 1 and 2 in the current study was not related to the primary gene encoding this factor, indicating that genetic variation outside of P1 is important for distinguishing isolates. Phylogenetic and population structure analyses of P1 gene sequences alone indicate that variation unrelated to P1 may be particularly important for subtyping of type 1 isolates.

Interestingly, only four clinical isolates were found to be closely related to the prototypical *M*. *pneumoniae* type 1 reference strain M129, suggesting that this genome is not representative of the majority of type 1 *M*. *pneumoniae* clinical isolates circulating worldwide. This substantiates a similar conclusion by Spuesens *et al*. [63] and calls into question the appropriateness of M129 as the type 1 reference genome. In contrast, FH was closely related to a large subgroup of type 2 isolates (n = 27). However, within type 2, the three subgroups were more equally divided compared to type 1, in which two of the subtypes consisted of only a few isolates each. Thus, additional reference strains, including at least representatives of each of the six subtypes, should be utilized for genomic comparisons. The importance of appropriately selected, high quality reference genomes cannot be overestimated.

Type 1 subpopulation 1N, which included isolates with three repeats at MLVA locus mpn16, also consisted of less than 10% of isolates examined in this study. Isolates with this particular locus pattern have been described previously outside of the United States, albeit much less frequently than other dominant MLVA types [56, 66, 67]. Many of the recently sequenced type 1 isolates were classified in the largest subgroup within this type and originated during the recent *M*. *pneumoniae* epidemic observed worldwide in 2010–2012 [55–57]. In the latter case, clonal expansion of outbreak strains would not be surprising. Additional analysis of type-specific sequence differences may inform the evolutionary history of *M*. *pneumoniae*, including the divergence of the two major lineages, the emergence of newer subtypes and clonal expansions.

Analysis of old isolates remains valuable to establish a historic baseline of strain variation. For example, the separation of four isolates from the southwestern United States in 2010–2012 may indicate a recently diverged subtype. But, continued monitoring is needed to assess the stability of this subpopulation and evaluate its geographic range. Within the type 2 group the strains identified as type 2v included not only isolates identified as this distinct subtype in our laboratory, but also two isolates from France, indicating that this subtype is not restricted to the United States. Similarly, isolates previously only categorized as type 2 in our laboratory appear to be more closely related to strain 309. Thus, type 2a seems to have a global distribution, and the number of isolates in this subgroup is similar to that of the FH-like (type 2Ref) group. The recent emergence of several subtypes and differences in geographic distribution may indicate ongoing divergence within the species or implicate patterns in transmission. Further investigation is needed to understand the impact of sequence changes in these isolates and the selective pressures underlying their emergence and potential spread.

We found that isolates collected during the same time period, even from a defined community during an epidemic period such as occurred in Colorado in 2013–2014 [60], West Virginia in 2011 [68], or Georgia in 2012 [69], separated primarily based on P1 type. This supports the polyclonal nature of *M*. *pneumoniae* circulating at any given time [69–75]. In contrast, isolates obtained from very close contacts, such as household members or residents of a long-term care facility were closely related to one another. This finding is consistent with the transmission mechanism of *M*. *pneumoniae* requiring close contact between individuals [2]. Further investigation is needed to fully understand the dynamics of *M*. *pneumoniae* during outbreaks within confined populations and community-wide or larger epidemic periods.

Lluch-Senar *et al*. suggested separation of *M*. *pneumoniae* into nine subtypes, including four within type 1 and five within type 2 based on SNPs and indels [8]. The subgroups identified in our population structure analysis differ in both the number and composition of subtypes. This may be due to differences in sequencing, assembly, or analysis methods, or may simply be a result of the inclusion of a substantially higher number of isolates in our study, allowing a more refined investigation of within-clade relatedness. Still, the clinical or epidemiological significance of *M*. *pneumoniae* subtypes remains to be determined, and the accumulation of WGS data along with novel analysis approaches will likely continue to reveal the population structure. As this unfolds, it will become increasingly necessary to develop a standardized nomenclature for defining these subpopulations in order to enable global tracking of *M*. *pneumoniae* over time. This system should be flexible enough to accommodate additional strain divergence, which may occur or be uncovered at a later date.

This study has several limitations. First, short read sequencing data may be insufficient to resolve known repetitive regions within *M*. *pneumoniae*, leading to misassembly, over- or under-prediction of coding sequences and the inability to capture regions used in traditional typing schemas. The key to resolving repetitive elements in this *M*. *pneumoniae* study was the use of long sequencing reads generated on the PacBio platform. The subset of available complete closed sequences were assessed independently from incomplete genome sequences in order to confirm observations when the inherent limitations of short reads may have confounded the analysis. We used strict parameters for genome coverage and sequence read quality, as well as an orthologous clustering method to define the core of all 107 genomes included in this study. In comparison, the core defined using only closed genomes was approximately 30% larger, meaning a substantial number of genes were not considered in the larger analysis. This may be due to only a single genome lacking a SNP or genomic region, even if the absence was due to misassembly or lack of sequencing reads. Higher genome content in the analysis may have resulted in higher resolution, potentially even revealing additional subtypes. Still, the benefit of including the higher number of genomes as well as the confirmation of population structure in closed genomes only support the use of this limited core. Despite the large collection of genomes analyzed here, the number of isolates in several of the subgroups was small. Sequencing of additional isolates may improve the classification into these subtypes or identify novel subtypes. Further testing and development of the Random Forest model with additional isolates is necessary to create an executable application and to evaluate the relevance of SNPs identified as important for separating subtypes. This machine learning technique may also be useful for identifying genetic features associated with clinical outcomes or epidemiological trends.

The inclusion of 107 genomes, of which 67 were newly sequenced here, along with the combination of long and short read sequencing technologies and the use of a predictive modeling technique, makes this study the most comprehensive analysis of *M*. *pneumoniae* genomes to date. Type-specific SNPs and indels were found to contribute to an underlying population structure consisting of six distinct subtypes that was supported by phylogenetic analysis of the core genome as well as a Bayesian inference method. Our WGS analysis has resulted in a reliable model to classify *M*. *pneumoniae* isolates into these six subtypes without having to rerun genome assembly and *de novo* BAPS analysis using an ensemble decision tree based machine-learning method. The Random Forest application also allowed for identification of SNPs of highest importance for separating the various subtypes. In addition, we were able to develop a novel molecular diagnostic assay for detection and typing of *M*. *pneumoniae* [54]. These substantial outputs demonstrate the potential of WGS to advance clinical microbiology and epidemiology for an important, yet often underestimated, respiratory pathogen. From a public health perspective, WGS will fundamentally improve the ability to monitor circulation of various *M*. *pneumoniae* types as well as identify emergence of new variants or genetic features that may impact transmission or virulence.

## Supporting information

S1 Fig Analysis of genomic content relative to reference genomes FH or M129. BRIG analysis of genomic content in various genome representations of reference strains FH (A) and M129 (B) along with all type 1 and type 2 closed genomes generated using Pacific Biosciences RSII platform in the current study. Type 1, n = 10; type 2, n = 6.
(TIF)

S2 Fig Impact of number and quality of genomes on core genome size determination. Core genome size for closed genomes (n = 34; 642 core protein sequences) and all isolates (n = 107; 464 core protein sequences) are indicated by * and ‡, respectively.
(TIF)

S3 Fig Multiple sequence alignment of *mpn16* MLVA locus. Multiple sequence alignment of *mpn16* locus used in MLVA characterization of *M*. *pneumoniae* [20] depicting three repeats present in four isolates introduced in this study: EPC104 (SAMN05391749), EPC83 (SAMN05391746), EPC122 (SAMN05391751), and NM1 (SAMN05391736), along with M129. The *mpn*16 VNTR region of these four genomes was aligned to the M129 reference genome, a MLVA type 4572 isolate harboring two repeats at *mpn16*. This alignment revealed that all four isolates have three copies of tandemly repeated sequence in the *mpn*16 locus instead of the previously reported absence of repeats [70]. Review of the sequence electropherograms for MLVA typing of these isolates uncovered that the amplicon corresponding to the triple repeat at *mpn*16 (400 bp) was masked by an amplicon of nearly identical size (399 bp) that corresponded to five tandem repeats at the *mpn*14 locus.
(TIF)

S4 Fig Phylogenetic tree of core genome alignments identified in all isolates in the current study (n = 107) based on kSNP analysis. (TIF)

S5 Fig Population structure based on phylogenetic analysis and hierBAPS analysis of P1 gene sequence. (A) Phylogenetic tree based on 1900 bp amplicon in P1 gene (MPN141). (B) hierBAPS output using P1 gene sequence for 59 isolates having complete unbroken P1 gene sequence available.
(TIF)

S6 Fig Protein sequence differences resulting from non-synonymous SNPs in CARDS toxin gene (MPN372). (A) Type-specific non-synonymous SNP T1112G resulting in I371S substitution in all type 2 isolates. (B) Non-synonymous SNP C217T resulting in P73S substitution in isolates recovered from three individuals with *M*. *pneumoniae* infection residing within the same household.
(TIF)

S7 Fig Hierarchical Bayesian Analysis of Population Structure (hierBAPS). hierBAPS performed on (A) all isolates in the current study (n = 107) and (B) closed genomes only (n = 34).
(TIF)

S1 Table Isolate and genomic characteristics for all isolates included in the current study. (DOCX)

S2 Table NCBI BioProject, BioSample and SRA accession IDs for newly sequenced genomes and references used in current study. (DOCX)

S3 Table Summary of datasets used to identify differential genomic regions with Mauve alignment software. (DOCX)

S4 Table Presence/absence of feature lists (n = 659) for Random Forest input. (XLSX)

S5 Table Variant sites by genome position present in each list (n = 659) for Random Forest input. (XLSX)

S6 Table Pairwise comparison of representations of reference genomes of M129 and FH. (DOCX)

S7 Table Proteins present only in core genome of closed genomes. (XLSX)

S8 Table Allele differences (n = 520) between *M. pneumoniae* types 1 and 2. (XLSX)

S9 Table Feature lists containing >1 variant site ranked by importance based on lowest error in Random Forest model. (XLSX)

S1 Text Supplementary bioinformatics methods. (DOCX)
