# Correction: Comprehensive bioinformatics analysis of *Mycoplasma pneumoniae* genomes to investigate underlying population structure and type-specific determinants

**DOI:** 10.1371/journal.pone.0186030

**Published:** 2017-10-04

**Authors:** Maureen H. Diaz, Heta P. Desai, Shatavia S. Morrison, Alvaro J. Benitez, Bernard J. Wolff, Jason Caravas, Timothy D. Read, Deborah Dean, Jonas M. Winchell

Accession numbers for 8 assemblies included in this analysis are missing from S2 Table. These accession numbers are: MJIT00000000, MJIU00000000, MJIV00000000, MJIW00000000, MJIX00000000, MJIY00000000, MJIZ00000000, MJJA00000000. Please view the correct S2 Table below.

These accession numbers are also missing from the Data Availability statement. The correct statement is: All complete genome assemblies are available from the NCBI Genbank database (accession numbers CP017327, CP017328, CP017329, CP017330, CP017331, CP017332, CP017333, CP017334, CP017335, CP017336, CP017337, CP017338, CP017339, CP017340, CP017341, CP017342, CP017343, MJIT00000000, MJIU00000000, MJIV00000000, MJIW00000000, MJIX00000000, MJIY00000000, MJIZ00000000, MJJA00000000). All sequencing data is available from the NCBI Sequence Read Archive database (accession numbers SRR3924583, SRR3924584, SRR3924595, SRR3924606, SRR3924617, SRR3924628, SRR3924639, SRR3924647, SRR3924648, SRR3924649, SRR3924585, SRR3924586, SRR3924587, SRR3924588, SRR3924589, SRR3924590, SRR3924591, SRR3924592, SRR3924593, SRR3924594, SRR3924596, SRR3924597, SRR3924598, SRR3924599, SRR3924600, SRR3924601, SRR3924602, SRR3924603, SRR3924604, SRR3924605, SRR3924607, SRR3924608, SRR3924609, SRR3924610, SRR3924611, SRR3924612, SRR3924613, SRR3924614, SRR3924615, SRR3924616, SRR3924618, SRR3924619, SRR3924620, SRR3924621, SRR3924622, SRR3924623, SRR3924624, SRR3924625, SRR3924626, SRR3924627, SRR3924629, SRR3924630, SRR3924631, SRR3924632, SRR3924633, SRR3924634, SRR3924635, SRR3924636, SRR3924637, SRR3924638, SRR3924640, SRR3924641, SRR3924642, SRR3924643, SRR3924644, SRR3924645, SRR3924646).

## Supporting information

S2 Table NCBI BioProject, BioSample and SRA accession IDs for newly sequenced genomes and references used in current study. (DOCX)

## References

[1] Diaz MH, Desai HP, Morrison SS, Benitez AJ, Wolff BJ, Caravas J, et al (2017) Comprehensive bioinformatics analysis of *Mycoplasma pneumoniae* genomes to investigate underlying population structure and type-specific determinants. PLoS ONE 12(4): e0174701 https://doi.org/10.1371/journal.pone.0174701 28410368 10.1371/journal.pone.0174701 PMC5391922

