# Supplementary figures and images for: Comprehensive bioinformatics analysis of Mycoplasma pneumoniae genomes to investigate underlying population structure and type-specific determinants
Source: PLoS One. 2017 Apr 14;12(4):e0174701. doi: 10.1371/journal.pone.0174701 (PMC5391922; doi:10.1371/journal.pone.0174701)

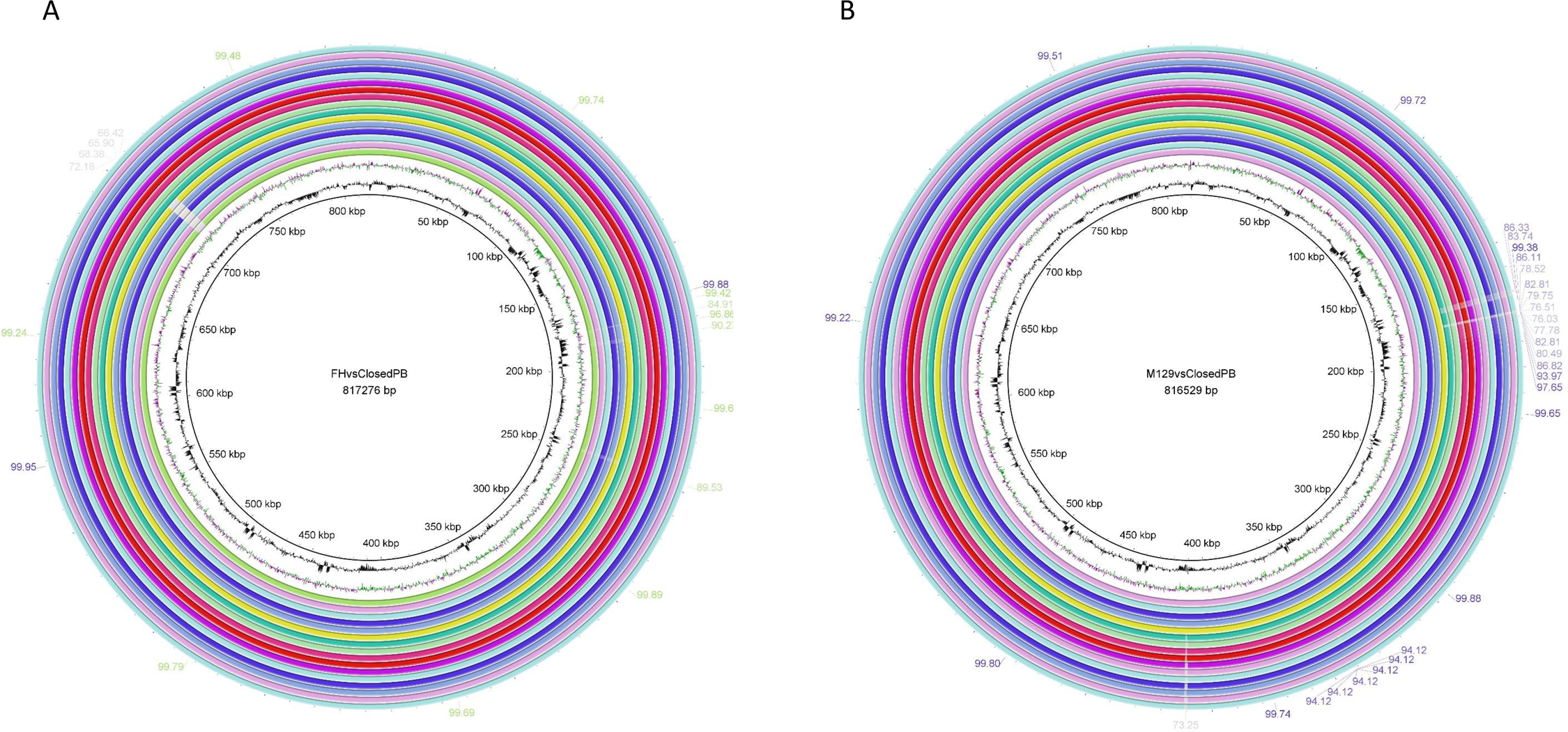

Supplement: S1 Fig — BRIG analysis of genomic content in various genome representations of reference strains FH (A) and M129 (B) along with all type 1 and type 2 closed genomes generated using Pacific Biosciences RSII platform in the current study. Type 1, n = 10; type 2, n = 6. (TIF) [file pone.0174701.s001.tif]

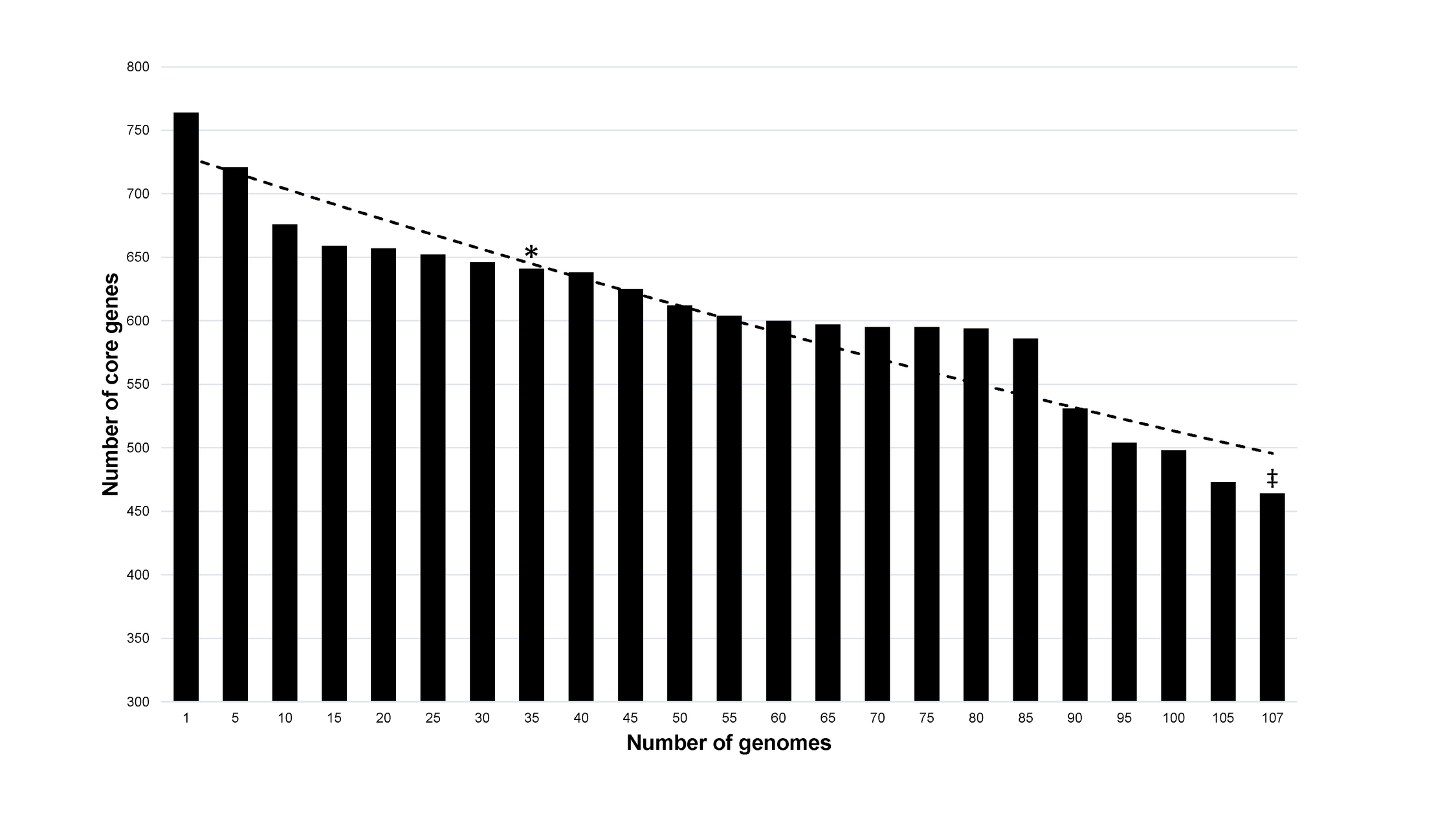

Supplement: S2 Fig — Core genome size for closed genomes (n = 34; 642 core protein sequences) and all isolates (n = 107; 464 core protein sequences) are indicated by * and ‡, respectively. (TIF) [file pone.0174701.s002.tif]

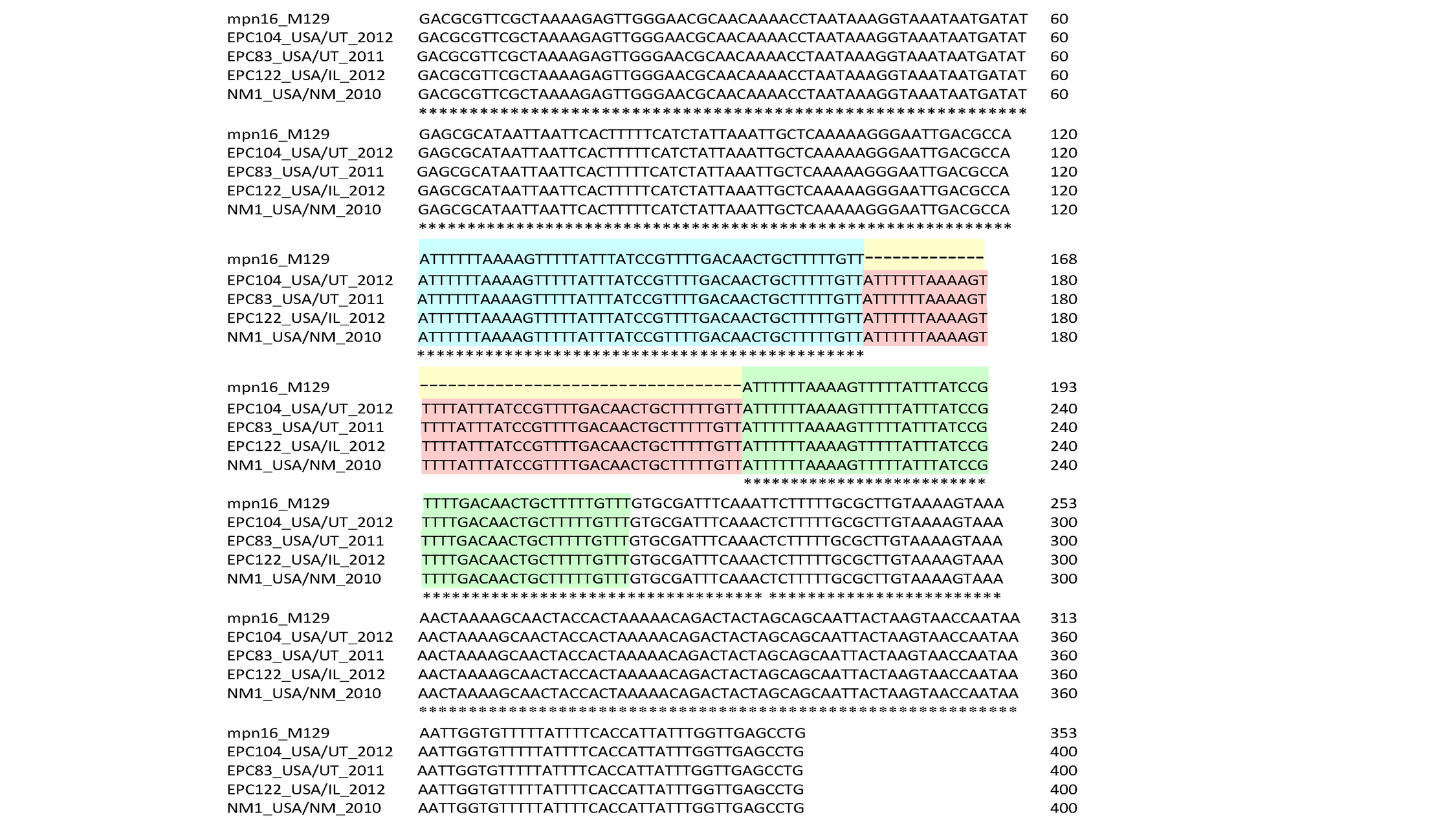

Supplement: S3 Fig — Multiple sequence alignment of mpn16 locus used in MLVA characterization of M. pneumoniae [20] depicting three repeats present in four isolates introduced in this study: EPC104 (SAMN05391749), EPC83 (SAMN05391746), EPC122 (SAMN05391751), and NM1 (SAMN05391736), along with M129. The mpn16 VNTR region of these four genomes was aligned to the M129 reference genome, a MLVA type 4572 isolate harboring two repeats at mpn16. This alignment revealed that all four isolates have three copies of tandemly repeated sequence in the mpn16 locus instead of the previously reported absence of repeats [70]. Review of the sequence electropherograms for MLVA typing of these isolates uncovered that the amplicon corresponding to the triple repeat at mpn16 (400 bp) was masked by an amplicon of nearly identical size (399 bp) that corresponded to five tandem repeats at the mpn14 locus. (TIF) [file pone.0174701.s003.tif]

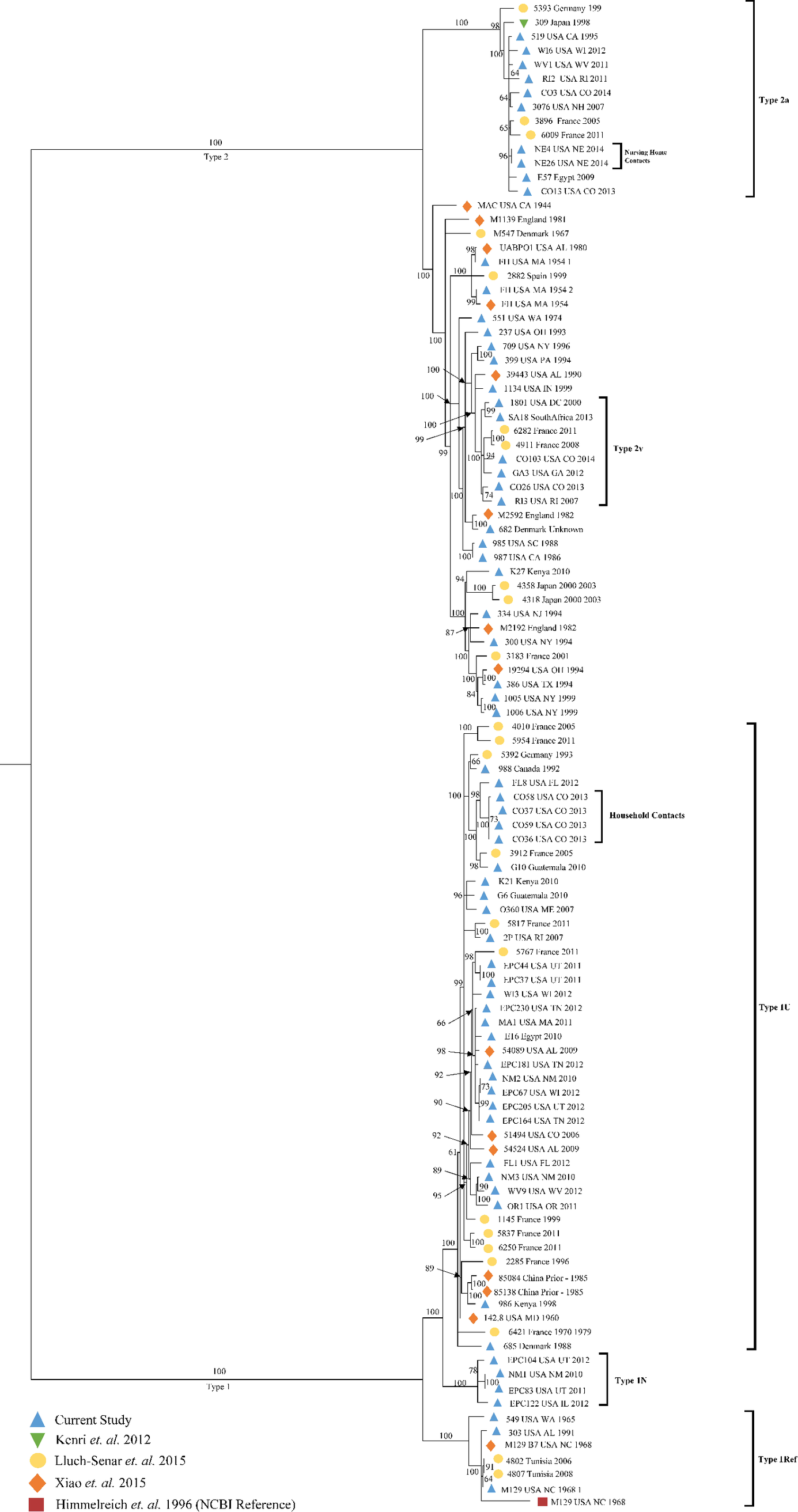

Supplement: S4 Fig — (TIF) [file pone.0174701.s004.tif]

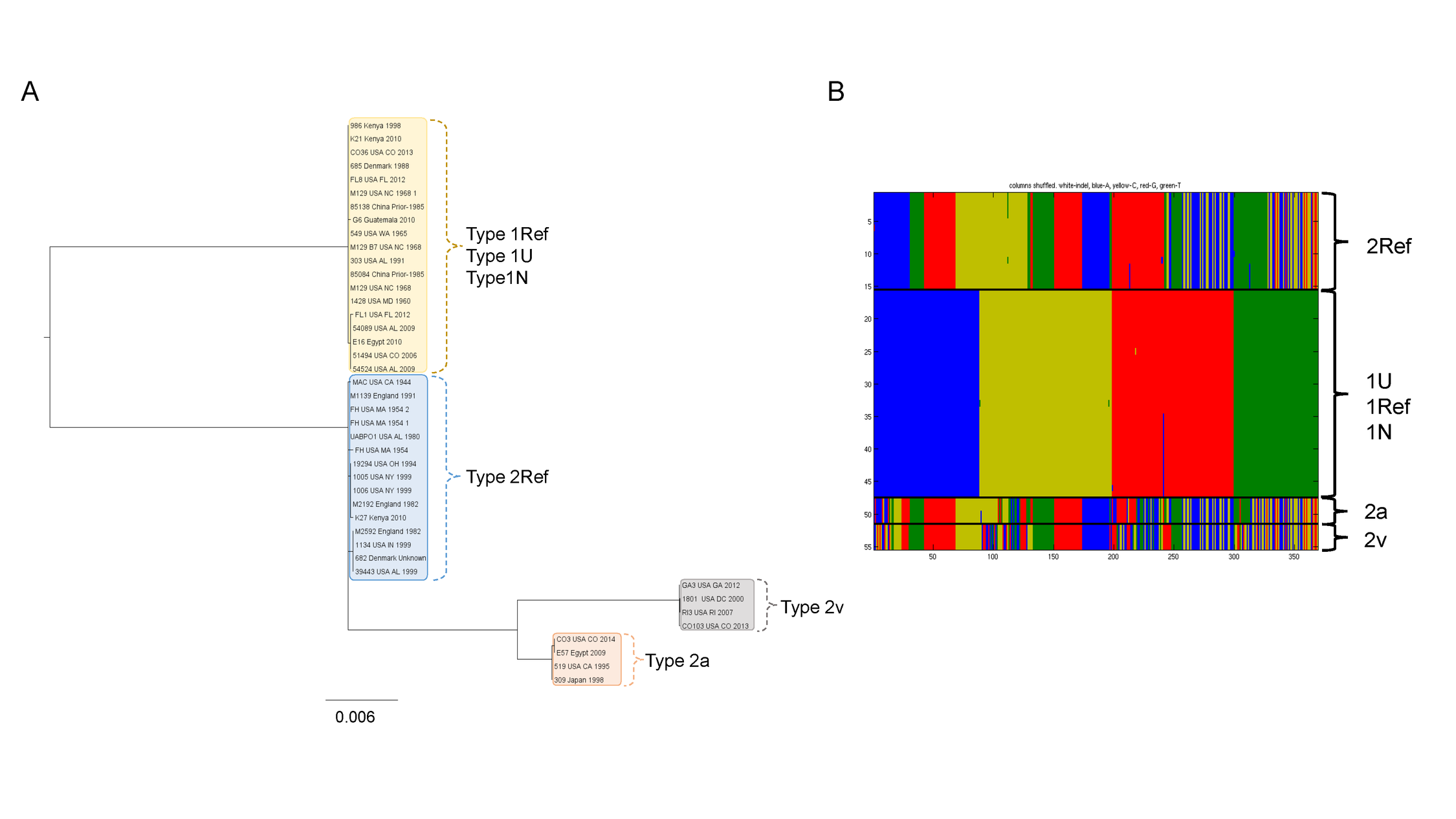

Supplement: S5 Fig — (A) Phylogenetic tree based on 1900 bp amplicon in P1 gene (MPN141). (B) hierBAPS output using P1 gene sequence for 59 isolates having complete unbroken P1 gene sequence available. (TIF) [file pone.0174701.s005.tif]

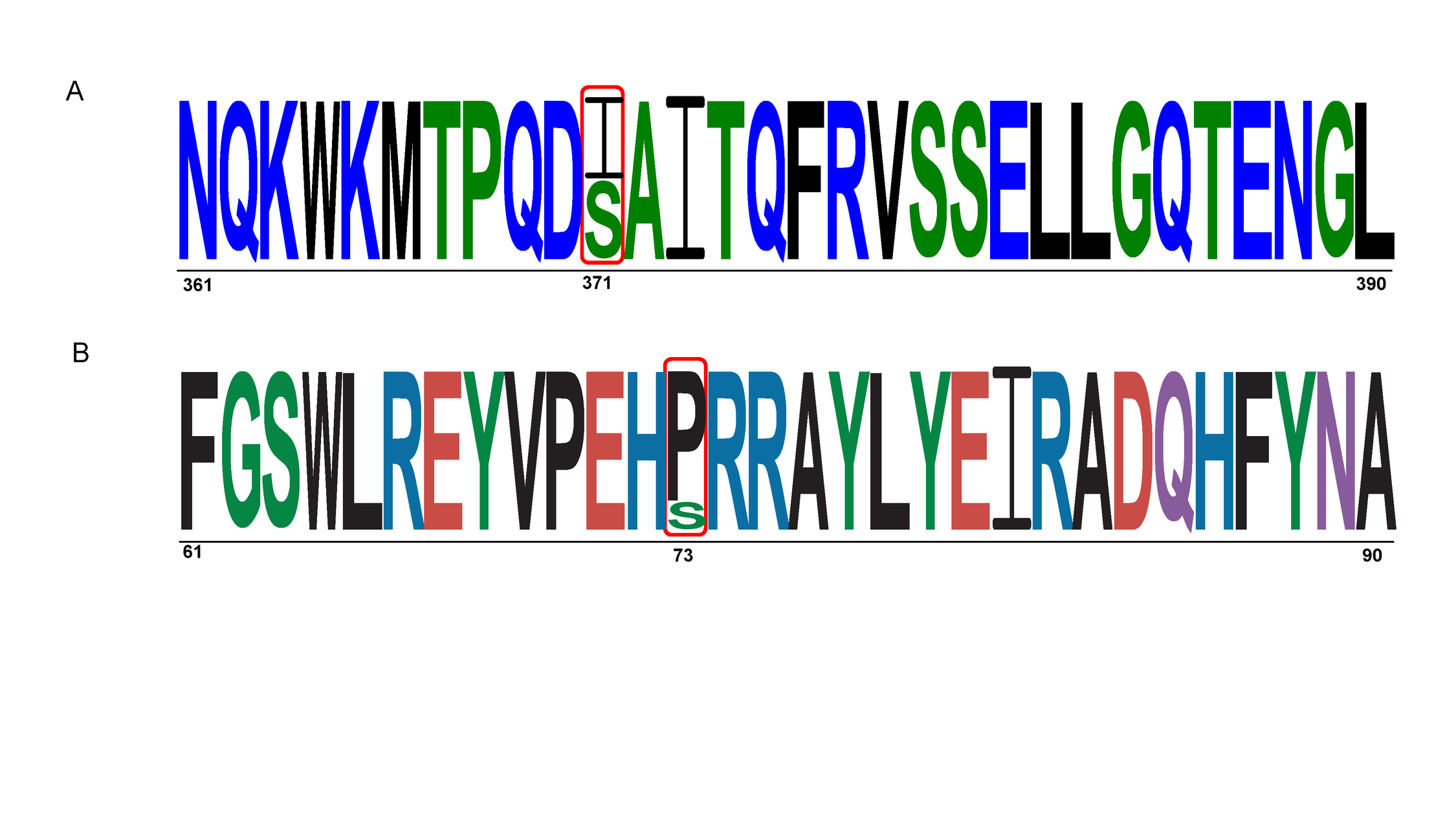

Supplement: S6 Fig — (A) Type-specific non-synonymous SNP T1112G resulting in I371S substitution in all type 2 isolates. (B) Non-synonymous SNP C217T resulting in P73S substitution in isolates recovered from three individuals with M. pneumoniae infection residing within the same household. (TIF) [file pone.0174701.s006.tif]

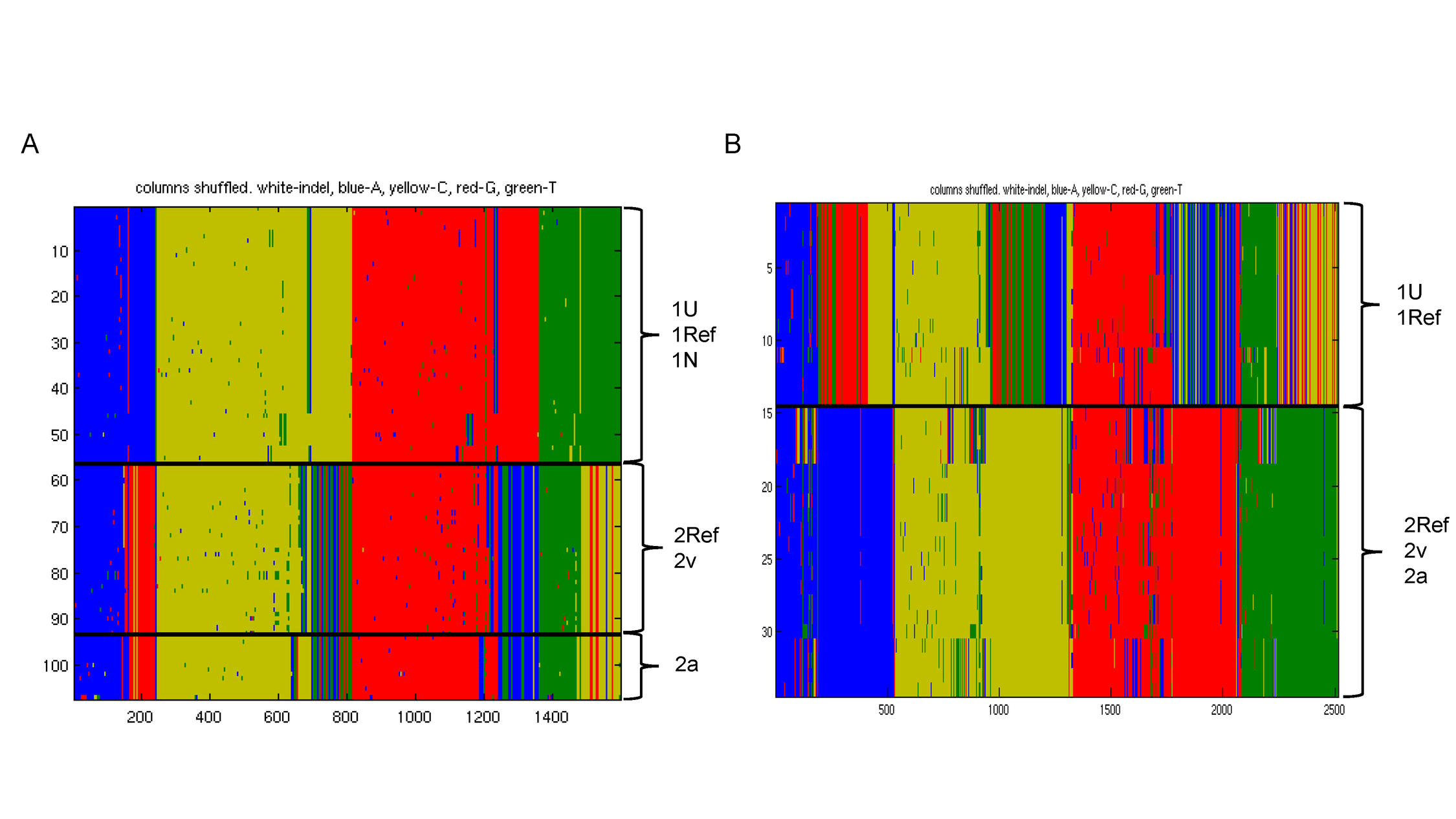

Supplement: S7 Fig — hierBAPS performed on (A) all isolates in the current study (n = 107) and (B) closed genomes only (n = 34). (TIF) [file pone.0174701.s007.tif]
