# Supplementary material for: Comprehensive bioinformatics analysis of Mycoplasma pneumoniae genomes to investigate underlying population structure and type-specific determinants
Source: PLoS One. 2017 Apr 14;12(4):e0174701. doi: 10.1371/journal.pone.0174701 (PMC5391922; doi:10.1371/journal.pone.0174701)
Supplement: S1 Table — (DOCX) [file pone.0174701.s008.docx]

**S1 Table. Isolate and genomic characteristics for all isolates included in the current study.**

| Isolates | Origin | Year | P1 Genotype | MLVA type | Macrolide Susceptibility | | Number of reads | GC content | Number of Contigs | Largest Contig | N50 | Genome coverage | Predicted genes | Study |
| --- | --- | --- | --- | --- | --- | --- | --- | --- | --- | --- | --- | --- | --- | --- |
| CO37 | Colorado | 2013 | 1 | 4572 | | S | 15717832^1^ | 41 | 21 | 166103 | 96004 | 3366.7 | 766 | This |
| OR1 | Oregon | 2011 | 1 | 4572 | | R | 17282718^1^ | 40 | 30 | 119652 | 46808 | 3701.9 | 767 | This |
| SA18 | South Africa | 2012 | 2V | 3662 | | S | 16797452^1^ | 41 | 30 | 200086 | 56851 | 3598 | 756 | This |
| FH 1965^4^ | Massachusetts | 1965 | 2 | 3662 | | S | 2074230^1^, | 41 | 2 | 675977 | 675977 | 139.99^*^ | 768 | This |
|  |  |  |  |  |  |  | 63521^2^ |  |  |  |  |  |  |  |
| FH 2009^4, 7^ | Massachusetts | 2009^3^ | 2 | 3662 | | S | 2729324^1^, | 39 | 1 | 823345 | 823345 | 99.65 | 758 | This |
|  |  |  |  |  |  |  | 38894^2^ |  |  |  |  |  |  |  |
| 685^7^ | Denmark | 1988 | 1 | 4572 | | R | 2611158^1^, | 39 | 1 | 827698 | 827698 | 90.61 | 753 | This |
|  |  |  |  |  |  |  | 55841^2^ |  |  |  |  |  |  |  |
| 988 | Canada | 1992 | 1 | 4572 | | S | 2568706^1^ | 39 | 7 | 642016 | 642016 | 550.2 | 758 | This |
| E57 | Egypt | 2009 | 2 | 3562 | | S | 4911048^1^, | 40 | 1 | 829,068 | 829,068 | 102.1 | 765 | This |
|  |  |  |  |  |  |  | 53705^2^ |  |  |  |  |  |  |  |
| G10 | Guatemala | 2010 | 1 | 4672 | | S | 3206836^1^ | 40 | 12 | 466802 | 466802 | 686.9 | 754 | This |
| NM3 | New Mexico | 2010 | 1 | 4572 | | R | 2402364^1^ | 41 | 5 | 340396 | 282536 | 514.6 | 759 | This |
| 549^7^ | Washington | 1965 | 1 | 4572 | | S | 3191606^1^, | 41 | 1 | 826463 | 826463 | 98.69 | 757 | This |
|  |  |  |  |  |  |  | 46447^2^ |  |  |  |  |  |  |  |
| WV9 | W. Virginia | 2012 | 1 | 4572 | | R | 2465220^1^ | 41 | 2 | 604348 | 604348 | 528 | 750 | This |
| FL8^7^ | Florida | 2012 | 1 | 4572 | | S | 2530190^1^, | 41 | 1 | 820984 | 820984 | 100.43 | 758 | This |
|  |  |  |  |  |  |  | 32640^2^ |  |  |  |  |  |  |  |
| 986 | Kenya | 1998 | 1 | 4572 | | S | 2612290^1^, | 41 | 4 | 313359 | 254024 | 114.63 | 779 | This |
|  |  |  |  |  |  |  | 29168^2^ |  |  |  |  |  |  |  |
| K21 | Kenya | 2010 | 1 | 4572 | | S | 2781858^1^, | 41 | 2 | 811423 | 811423 | 131.06 | 787 | This |
|  |  |  |  |  |  |  | 44205^2^ |  |  |  |  |  |  |  |
| G6 | Guatemala | 2010 | 1 | 4572 | | S | 2935828^1^, | 41 | 4 | 380268 | 212503 | 117.23 | 769 | This |
|  |  |  |  |  |  |  | 45486^2^ |  |  |  |  |  |  |  |
| E16^7^ | Egypt | 2010 | 1 | 4572 | | S | 3152010^1^, | 40 | 1 | 826912 | 826912 | 145.1^*^ | 758 | This |
|  |  |  |  |  |  |  | 38341^2^ |  |  |  |  |  |  |  |
| 303 | Alabama | 1991 | 1 | 4572 | | S | 3190118^1^, | 41 | 6 | 533215 | 533215 | 90.25 | 787 | This |
|  |  |  |  |  |  |  | 49138^2^ |  |  |  |  |  |  |  |
| FL1^7^ | Florida | 2012 | 1 | 4672 | | S | 2379846^1^, 33391^2^ | 41 | 1 | 822592 | 822592 | 94.05 | 756 | This |
| MA1 | Massachusetts | 2011 | 1 | 4572 | | S | 2731742^1^ | 41 | 32 | 195477 | 188384 | 585.1 | 750 | This |
| K27^7^ | Kenya | 2010 | 2 | 3662 | | S | 3398314^1^, | 40 | 1 | 823783 | 823783 | 102.1 | 758 | This |
|  |  |  |  |  |  |  | 32833^2^ |  |  |  |  |  |  |  |
| CO103^7^ | Colorado | 2013 | 2V | 3662 | | S | 1935756^1^, | 40 | 1 | 827908 | 827908 | 99.24 | 749 | This |
|  |  |  |  |  |  |  | 48196^2^ |  |  |  |  |  |  |  |
| GA3 | Georgia^5^ | 2012 | 2V | 3662 | | S | 2636720^1^, | 40 | 1 | 825033 | 825033 | 96.9 | 746 | This |
|  |  |  |  |  |  |  | 41078^2^ |  |  |  |  |  |  |  |
| 1005 | New York | 1999 | 2 | 3662 | | S | 3569170^1^, | 40 | 2 | 800059 | 800059 | 149.4^*^ | 758 | This |
|  |  |  |  |  |  |  | 58372^2^ |  |  |  |  |  |  |  |
| 1006^7^ | New York | 1999 | 2 | 3662 | | R | 2878870^1^ , 63529^2^ | 40 | 1 | 824365 | 824365 | 97.56 | 751 | This |
| 987 | California | 1986 | 2 | 3662 | | S | 4179004^1^ | 40 | 9 | 393259 | 188616 | 895.1 | 750 | This |
| 1134^7^ | Indiana | 1999 | 2 | 3662 | | S | 2750042^1^ , 38998^2^ | 40 | 1 | 826768 | 826768 | 118.22 | 745 | This |
| RI2 | Rhode Island | 2011 | 2 | 3562 | | S | 2358714^1^ | 40 | 14 | 297329 | 188840 | 505.2 | 766 | This |
| 985 | S. Carolina | 1988 | 2 | 3662 | | S | 2595710^1^ | 40 | 10 | 393490 | 188823 | 556 | 764 | This |
| 519^7^ | California | 1995 | 2 | 3562 | | S | 2500824^1^, | 40 | 1 | 830076 | 830076 | 101.4 | 765 | This |
|  |  |  |  |  |  |  | 28329^2^ |  |  |  |  |  |  |  |
| 237 | Ohio | 1993 | 2 | 3662 | | S | 2023556^1^ | 40 | 15 | 338947 | 188780 | 433.4 | 770 | This |
| 682 | Denmark | N/A | 2 | 3562 | | S | 2728894^1^, | 40 | 3 | 552264 | 552264 | 131.63^*^ | 777 | This |
|  |  |  |  |  |  |  | 49285^2^ |  |  |  |  |  |  |  |
| RI3^7^ | Rhode Island | 2007 | 2V | 3662 | | S | 8590310^1^, | 40 | 1 | 825063 | 825063 | 55.99^*^ | 746 | This |
|  |  |  |  |  |  |  | 37631^2^ |  |  |  |  |  |  |  |
| 1801^7^ | Washington DC | 2000 | 2V | 3662 | | S | 2948326^1^, | 40 | 1 | 826961 | 826961 | 245.95^*^ | 747 | This |
|  |  |  |  |  |  |  | 40743^2^ |  |  |  |  |  |  |  |
| 334 | New Jersey | 1994 | 2 | 3662 | | S | 2188654^1^ | 40 | 10 | 393367 | 188572 | 468.8 | 767 | This |
| 3076 | New Hampshire | 2007 | 2 | 3562 | | S | 6025988^1^ | 40 | 17 | 233661 | 95694 | 1290.8 | 770 | This |
| 300 | New York | 1994 | 2 | 3662 | | S | 2799318^1^ | 40 | 15 | 365989 | 153480 | 599.6 | 759 | This |
| 709 | New York | 1996 | 2 | 3562 | | S | 2968974^1^ | 40 | 11 | 373997 | 188992 | 635.9 | 756 | This |
| O-360 | Maine | 2007 | 1 | 4572 | | S | 3313408^1^ | 41 | 4 | 459602 | 459602 | 709.7 | 752 | This |
| 399 | Pennsylvania | 1994 | 2 | 3562 | | S | 3438984^1^ | 40 | 8 | 338932 | 180467 | 736.6 | 744 | This |
| 2P | Rhode Island | 2007 | 1 | 4572 | | R | 3436248^1^ | 41 | 4 | 445159 | 445159 | 736 | 754 | This |
| 386 | Texas | 1994 | 2 | 3562 | | S | 3788942^1^ | 40 | 13 | 382155 | 153283 | 811.6 | 759 | This |
| 551 | Washington | 1974 | 2 | 3662 | | S | 3171374^1^ | 40 | 8 | 393132 | 262892 | 679.3 | 754 | This |
| WI3 | Wisconsin | 2012 | 1 | 4572 | | S | 3048788^1^ | 41 | 1 | 817430 | 817430 | 653 | 754 | This |
| WI6 | Wisconsin | 2012 | 2 | 3562 | | S | 3203608^1^ | 40 | 13 | 297376 | 188773 | 686.2 | 770 | This |
| WV1 | W. Virginia | 2011 | 2 | 3562 | | S | 3314968^1^ | 40 | 11 | 212767 | 153346 | 710.1 | 765 | This |
| NE4 | Nebraska | 2014 | 2 | 3562 | | S | 2630864^1^ | 40 | 13 | 297639 | 189025 | 563.5 | 765 | This |
| NE26 | Nebraska | 2014 | 2 | 3562 | | S | 2931652^1^ | 40 | 14 | 188844 | 153345 | 628 | 763 | This |
| NM1 | New Mexico | 2010 | 1 | 4572 | | S | 2108882^1^ | 40 | 7 | 425014 | 425014 | 451.7 | 759 | This |
| NM2 | New Mexico | 2010 | 1 | 4572 | | R | 2181184^1^ | 40 | 6 | 220326 | 206518 | 467.2 | 760 | This |
| CO13 | Colorado | 2013 | 2 | 3562 | | S | 2734214^1^ | 41 | 12 | 213243 | 153337 | 585.7 | 767 | This |
| CO59 | Colorado | 2013 | 1 | 4572 | | S | 3369502^1^ | 40 | 7 | 372211 | 113371 | 721.7 | 756 | This |
| CO36 | Colorado | 2013 | 1 | 4572 | | S | 793818^1^, | 41 | 2 | 752508 | 752508 | 140.32^*^ | 776 | This |
|  |  |  |  |  |  |  | 46458^2^ |  |  |  |  |  |  |  |
| CO3^7^ | Colorado | 2014 | 2 | 3562 | | S | 3237502^1^ , 47476^2^ | 40 | 1 | 825203 | 825203 | 96.74 | 768 | This |
| CO58 | Colorado | 2013 | 1 | 4572 | | S | 3397094^1^ | 41 | 6 | 206426 | 165442 | 727.7 | 759 | This |
| CO26 | Colorado | 2013 | 2V | 3662 | | S | 3602084^1^ | 40 | 12 | 339070 | 153262 | 771.6 | 749 | This |
| M129^4, 7^ | North Carolina | 2002^3^ | 1 | 4572 | | S | 2886504^1^, | 41 | 1 | 827044 | 827044 | 103.12 | 757 | This |
|  |  |  |  |  |  |  | 43383^2^ |  |  |  |  |  |  |  |
| EPC205 | Utah | 2012 | 1 | 4572 | | R | 3611130^1^ | 40 | 6 | 412315 | 412315 | 773.5 | 756 | This |
| EPC83 | Utah | 2011 | 1 | 4570 | | S | 2650402^1^ | 39 | 7 | 372199 | 208045 | 567.7 | 756 | This |
| EPC67 | Tennessee | 2012 | 1 | 4572 | | R | 3098600^1^ | 40 | 5 | 372120 | 277175 | 663.7 | 757 | This |
| EPC181 | Tennessee | 2012 | 1 | 4572 | | S | 3345930^1^ | 40 | 14 | 323206 | 112966 | 716.7 | 759 | This |
| EPC104 | Utah | 2012 | 1 | 5570 | | S | 3010998^1^ | 40 | 13 | 424883 | 424883 | 645 | 757 | This |
| EPC37 | Utah | 2011 | 1 | 4072 | | S | 2846942^1^ | 40 | 10 | 430206 | 430206 | 609.8 | 758 | This |
| EPC122 | Illinois | 2012 | 1 | 4570 | | S | 3338458^1^ | 40 | 8 | 206683 | 96030 | 715.1 | 752 | This |
| EPC164 | Tennessee | 2012 | 1 | 4572 | | R | 2957002^1^ | 39 | 5 | 372378 | 174383 | 633.4 | 755 | This |
| EPC44 | Utah | 2011 | 1 | 4572 | | S | 3572762^1^ | 39 | 13 | 372169 | 156775 | 765.3 | 760 | This |
| EPC230 | Tennessee | 2012 | 1 | 4572 | | S | 2895822^1^ | 39 | 10 | 372870 | 156807 | 620.3 | 760 | This |
| M129^4, 7^ | North Carolina | 1968 | 1 |  | | S |  |  | 1 |  |  |  | 764 | Himme-lreich  *et.al* |
| 309^7^ | Japan | 1998 | 2a |  | | S |  |  | 1 |  |  |  | 776 | Kenri *et. al* |
| M129-B7^4, 7^ | North Carolina | 1968 | 1 |  | | S |  |  | 1 |  |  |  | 758 | Xiao *et. al* |
| 142.8^7^ | Maryland | 1960 | 1 |  | | S |  |  | 1 |  |  |  | 757 | Xiao *et. al* |
| 51494^7^ | Colorado | 2006 | 1 |  | | S |  |  | 1 |  |  |  | 757 | Xiao *et. al* |
| 54089^7^ | Alabama | 2009 | 1 |  | | R |  |  | 1 |  |  |  | 756 | Xiao *et. al* |
| 54524^7^ | Alabama | 2009 | 1 |  | | S |  |  | 1 |  |  |  | 756 | Xiao *et. al* |
| 85084^7^ | China | Prior to 1985 | 1 |  | | S |  |  | 1 |  |  |  | 756 | Xiao *et. al* |
| 85138^7^ | China | Prior to 1985 | 1 |  | | S |  |  | 1 |  |  |  | 756 | Xiao *et. al* |
| FH^4, 7^ | Massachusetts | 1954 | 2 |  | | S |  |  | 1 |  |  |  | 759 | Xiao *et. al* |
| 19294^7^ | Ohio | 1994 | 2 |  | | S |  |  | 1 |  |  |  | 756 | Xiao *et. al* |
| 39443^7^ | Alabama | 1999 | 2 |  | | S |  |  | 1 |  |  |  | 750 | Xiao *et. al* |
| M1139^7^ | England | 1981 | 2 |  | | S |  |  | 1 |  |  |  | 753 | Xiao *et. al* |
| M2192^7^ | England | 1982 | 2 |  | | S |  |  | 1 |  |  |  | 754 | Xiao *et. al* |
| M2592^7^ | England | 1982 | 2 |  | | S |  |  | 1 |  |  |  | 755 | Xiao *et. al* |
| MAC^7^ | California | 1944 | 2 |  | | S |  |  | 1 |  |  |  | 757 | Xiao *et. al* |
| UAB PO1^7^ | Alabama | 1980 | 2 |  | | S |  |  | 1 |  |  |  | 759 | Xiao *et. al* |
| 1145 | France | 1999 | 1 | 4572 | | S | 1356570 | 39 | 26 | 472942 | 472942 | 149.44 | 758 | Lluch-Senar *et. al* |
| 2285 | France | 1996 | 1 | 4572 | | S | 1356924 | 39 | 33 | 223296 | 127468 | 149.48 | 755 | Lluch-Senar *et. al* |
| 2882 | Spain | 1999 | 2 | 3662 | | S | 1357090 | 39 | 61 | 328707 | 97475 | 149.50 | 762 | Lluch-Senar *et. al* |
| 3163 | France | 2001 | 2 | 3662 | | S | 1357174 | 39 | 53 | 224815 | 192126 | 149.51 | 762 | Lluch-Senar *et. al* |
| 3896 | France | 2005 | new type | 3562 | | S | 1357228 | 39 | 53 | 192088 | 223813 | 149.51 | 752 | Lluch-Senar *et. al* |
| 3912 | France | 2005 | 1 | 4572 | | S | 1356142 | 39 | 39 | 406710 | 126920 | 149.39 | 763 | Lluch-Senar *et. al* |
| 4010 | France | 2005 | 1 | 3572 | | S | 1356800 | 39 | 35 | 529777 | 529777 | 149.46 | 762 | Lluch-Senar *et. al* |
| 4318 | Japan | 2000-2003 | 2 | 3662 | | S | 1356728 | 39 | 44 | 450545 | 450545 | 149.46 | 765 | Lluch-Senar *et. al* |
| 4358 | Japan | 2000-2003 | 2 | 3662 | | R | 1356868 | 39 | 59 | 132481 | 66856 | 149.47 | 767 | Lluch-Senar *et. al* |
| 4802 | Tunisia | 2006 | 1 | 4572 | | S | 1354224 | 39 | 39 | 235609 | 223227 | 149.18 | 751 | Lluch-Senar *et. al* |
| 4807 | Tunisia | 2008 | 1 | 4572 | | S | 1356948 | 39 | 54 | 162289 | 132248 | 149.48 | 751 | Lluch-Senar *et. al* |
| 4911 | France | 2008 | 2 | 3662 | | S | 1356352 | 39 | 53 | 224003 | 192139 | 149.41 | 757 | Lluch-Senar *et. al* |
| 5392 | Germany | 1993 | 1a | 4572 | | S | 1357358 | 39 | 37 | 304313 | 162702 | 149.53 | 756 | Lluch-Senar *et. al* |
| 5393 | Germany | 1991 | 2a | 3562 | | S | 1357204 | 39 | 45 | 434729 | 434729 | 149.51 | 767 | Lluch-Senar *et. al* |
| 5767 | France | 2011 | 1 | 4572 | | S | 1355902 | 39 | 33 | 224519 | 246492 | 149.36 | 755 | Lluch-Senar *et. al* |
| 5817 | France | 2011 | 1 | 4572 | | S | 1354164 | 39 | 31 | 310181 | 187146 | 149.17 | 753 | Lluch-Senar *et. al* |
| 5837 | France | 2011 | 1 | 4572 | | S | 1356420 | 39 | 47 | 398536 | 148180 | 149.42 | 763 | Lluch-Senar *et. al* |
| 5954 | France | 2011 | 1 | 4572 | | R | 1356044 | 39 | 39 | 209458 | 126855 | 149.38 | 753 | Lluch-Senar *et. al* |
| 6009 | France | 2011 | 2a | 3562 | | S | 1356536 | 39 | 72 | 223640 | 192292 | 149.43 | 771 | Lluch-Senar *et. al* |
| 6250 | France | 2011 | 1 | 4572 | | S | 1355582 | 39 | 40 | 371654 | 218818 | 149.33 | 767 | Lluch-Senar *et. al* |
| 6282^6^ | France | 2011 | 2 | 4572 | | S | 1356364 | 39 | 32 | 401703 | 251684 | 149.42 | 747 | Lluch-Senar *et. al* |
| 6421 | France | 1970's | 1 | 4572 | | S | 1356612 | 39 | 37 | 345924 | 126622 | 149.44 | 774 | Lluch-Senar *et. al* |
| M547 | Denmark | 1967 | 2 | 3662 | | S | 1356088 | 39 | 53 | 226007 | 192283 | 149.39 | 762 | Lluch-Senar *et. al* |

^1^ Denotes Illumina MiSeq Read Count

**^2^** Denotes Pacific BioScience RSII Read Count

**^3^**Year acquired from ATCC

**^4^***M. pneumoniae* type strain

^5^Acquired from Country U.S.A

^6^ Lluch-Senar *et. al* had previously reported P1 typing of strain 6282 as Type 1; however, the authors acknowledged discrepancies and classified as Type 2 strain.

^7^ Included in analysis of closed genomes only (n=34)

N/A – Not available

S – Sensitive, R - Resistant

**^*^**Error Corrected with Illumina Reads, if coverage dropped lower, contigs would break.

^Red^ denote that Pacific BioSciences sequencing data was generated for that specific isolate.
