# Supplementary material for: Comprehensive bioinformatics analysis of Mycoplasma pneumoniae genomes to investigate underlying population structure and type-specific determinants
Source: PLoS One. 2017 Apr 14;12(4):e0174701. doi: 10.1371/journal.pone.0174701 (PMC5391922; doi:10.1371/journal.pone.0174701)
Supplement: S3 Table — (DOCX) [file pone.0174701.s010.docx]

**S3 Table. Summary of datasets used to identify differential genomic regions with Mauve alignment software.**

| **Dataset** | **1** | **2** | **3** | **4** | **5** | **6** | **7** |
| --- | --- | --- | --- | --- | --- | --- | --- |
| Genomes included | M129  M129-B7  FH  309 | All Type 1  M129  M129-B7 | All Type 2  FH  309 | All Type 1  M129  M129-B7  FH  309 | All Type 2  M129  M129-B7  FH  309 | All Type 2V  FH  309 | All (n=107) |
