# Supplementary material for: Comprehensive bioinformatics analysis of Mycoplasma pneumoniae genomes to investigate underlying population structure and type-specific determinants
Source: PLoS One. 2017 Apr 14;12(4):e0174701. doi: 10.1371/journal.pone.0174701 (PMC5391922; doi:10.1371/journal.pone.0174701)
Supplement: S6 Table — (DOCX) [file pone.0174701.s013.docx]

**S6 Table. Pairwise comparison of representations of reference genomes of M129 and FH.**

| **Genome (Strain_ sequencing method)** | **Accession number** | **Length** | **Genes** | **CDS** | **tRNA** | **tmRNA** | **SNPs^1^** |
| --- | --- | --- | --- | --- | --- | --- | --- |
| M129-B7_MiSeq^2^ | NC_020076.2 | 816451 | 794 | 793 | 36 | 1 | N/A |
| M129_Sanger | NC_000912.1 | 816394 | 800 | 763 | 36 | 1 | 183 |
| M129_PacBioRSII | CP017343 | 816529 | 793 | 756 | 36 | 1 | 76 |
| FH_MiSeq^2^ | NZ_CP010546.1 | 817207 | 795 | 758 | 36 | 1 | N/A |
| FH_454 | NC_017504.1 | 811088 | 804 | 767 | 36 | 1 | 80 |
| FH_2009_PacBioRSII | CP017327 | 817276 | 794 | 757 | 36 | 1 | 24 |

^1^Relative to type-specific reference genome

^2^Used as reference for pairwise comparison
