# Supplementary material for: Comprehensive bioinformatics analysis of Mycoplasma pneumoniae genomes to investigate underlying population structure and type-specific determinants
Source: PLoS One. 2017 Apr 14;12(4):e0174701. doi: 10.1371/journal.pone.0174701 (PMC5391922; doi:10.1371/journal.pone.0174701)
Supplement: S1 Text — (DOCX) [file pone.0174701.s017.docx]

**S1 Text**

**Supplementary Bioinformatics Methods**

**Iterative Assembly Process**

VelvetOptimiser.pl is a “wrapper script” that can take a range of odd hash values to test on velvet’s assembler, and outputs the most optimized assembly for given input data [1]. However, running it for an optimized range could be inflexible for converging the value in an automated pipeline because this range could be anything from lowest possible k-mer to maximum read-length, and velvetOptimiser would need to be run multiple times. VelvetK.pl was utilized to avoid this by finding the best optimal k-mer size as an input for velvetOptimiser range. This script (velvetK.pl) outputs a single k-mer value, which finds the starting hash value (-s) by subtracting 5 from k-mer value and finds the end hash value (-e) by adding 5 to k-mer value in the pipeline used for this analysis.

**Read Alignment and SNP Optimizations**

The cleansed sequencing reads for each newly sequenced isolate were mapped using Bowtie2 with default parameters [2] to the respective type genome reference;  type 1 isolates were mapped to *Mycoplasma pneumoniae* M129 (Accession: NC_000912.1) and type 2 isolates to *M. pneumoniae* FH (Accession: NZ_CP010546.1). Samtools v0.1.18[3] was used to convert sam files to bam files and index each of the alignment files, and mpileup command was used to generate a pileup file for each isolate. The pileup file served as a guide to confirm any irregular alignments at repeat regions in newly sequenced genomes as well as heterozygous base calls at a position for correctness and alignment confidence.  SNP calls were made using Freebayes v0.9.21. To ensure the quality of variants, some of the parameters were changed from default. Since there are a very few reported SNP sites or insertion/deletion regions in *M. pneumoniae*, we restricted our analysis to reference based SNP identifications only using –i parameter in Freebayes. To confirm high quality and confident alternate allele calls, the following parameters for Freebayes were changed: ploidy –p was set to 1, alternate allele observation parameter –c/--min-alternate-count to 95, minimum coverage required to assess the position was set to 100 with –min-coverage parameter, minimum base quality for supporting position was set to 25, and minimum mapping quality for alignments was set to 15.  Despite the stringency in SNP calls, there were positive variant calls with a very low likelihood quality score, indicating a low probability of these calls being true; these were classified as false positives and were removed from the remainder of the analysis.

**PanOct Sequence Identity and Coverage Cutoffs**

Orthologous clusters were defined with all sequences in a cluster as necessarily having a shared sequence identity and gene length coverage > 75%. The *in silico* results were verified using previously established detection and characterization markers for *M. pneumoniae,* including: the Community-Acquired Respiratory Distress Syndrome (CARDS) toxin gene identified as a ADP-ribosylating toxin (locus tag MPN372), along with two other ATPase genes, ATP synthase subunit beta (locus tag MPN598) and lipoprotein (locus tag MPN588) [4]. Since sequence similarity is reportedly >99% between the type 1 and 2 as well as variants [5], we used high cutoff values of sequence identity and length coverage after trying a series of cluster experiments to find the optimal values. Once the clusters predicted by PanOCT included the known CARDS toxin protein as well as the previously mentioned other protein clusters consisting of all bacterial strains in study, the optimal values were selected. The presence of these genes in our core genome analysis served as a supportive benchmark to ensure that the parameters used in PanOCT were appropriate and that any potential novel findings were true genomic variations.

**Mauve Alignments Datasets**

For identifying regions that were present in all type 1 genomes compared to all type 2 genomes, seven datasets were created (Supplementary Table 8). Each dataset was used to perform an alignment using progressiveMauve for checking presence and absence of unique regions [6, 7]. If at least one genome was missing a region of interest, the region was not considered to be type-specific. Since these datasets included incomplete assemblies, it posed some limitations for analyzing these missing regions in an alignment for identification of type-specific regions.

**Core Genome Analysis – Closed Core**

To define a core genome for *M. pneumoniae*, groups were formed by randomly selecting five genomes to construct a set, thus making a total of 23 groups from 107 isolates. The core genome of each of the 23 groups was constructed using clusters that shared sequence identity and gene length coverage of > 75%. These groups were analyzed by comparing number of orthologous gene clusters in each group versus number of isolates in each group (S2 Fig). The clusters were created by adding closed genomes available in NCBI first, then adding 17 closed genomes sequenced in this study using Pacific Bioscience long sequencing read technology, and ultimately adding genomes obtained using Illumina 2 x 250 reads followed by Illumina 2 x 150 reads. The appropriate core genome of *M. pneumoniae* is expected to be around 595 genes; however, a sharp drop is seen when genome assemblies obtained from Illumina 2 x 150 kits published with SRA accession SRP061659 [8] were added. We performed this analysis to ensure that the genome sequencing was able to detect the majority, if not all, of the genetic components for our differential analysis. We noted that when a core genome is identified using draft genomes there is a potential to miss content that was not sequenced or captured in the sequencing library for that isolate. This shows the importance of using completely closed and characterized genomes in comparative genomic studies.

**Synonymous and non-synonymous mutation analysis**

While we identified several branches with a signature of adaptive evolution (ω > 1), the short branch lengths in the tree meant that most branches had insufficient observations to generate a reliable estimate of ω. We filtered these results to include only those branches for which we would predict at least two synonymous substitutions (S * dS ≥ 2). As a consequence of this filtering, our ability to detect adaptive evolution is restricted to the longest branches in our tree and the longest gene alignments.

After filtering out the lowest power estimates, only two gene alignments (ALA35790.1 GTP-Binding Protein Era and ALA36063.1 GTP-Binding Protein Era) contained branches which showed signs of adaptive evolution. We examined the alignment files for these genes and identified apparent homopolymer length polymorphisms in both genes which caused frame shifts near the C-terminus of encoded proteins. As the homopolymer length polymorphism is potentially due to a sequence assembly error and the frame shift actually represents a single mutational event (one single base deletion rather than multiple non-synonymous substitutions), we did not consider this to be clear evidence of adaptive evolution in these genes.

To detect genes which may show signs of relaxed purifying selection or adaptive pressure on only a subset of residues, we extended our investigation to genes with above average ω values. The majority of these gene alignments appeared to contain sequence assembly errors or possible miscalled orthology, however two gene alignments did not contain any obvious anomalies that would account for their elevated ω value. DNA Ligase (ALA35866.1) had a ω value of 0.3212 (S * *d*_S_ = 2.7, N * *d*_N_ =2.1) and DNA Polymerase III subunit alpha (ALA35550.1) had an ω value of 0.2746 (S * *d*_S_ = 2.0, N * *d*_N_ =1.2). The ω values listed correspond to the split between type 1 and type 2 *M. pneumoniae* isolates. While these ω values are below the 1.0 cutoff for confirmation of adaptive evolution, their above average rate of non-synonymous substitution suggests that purifying selection may be relaxed on these genes or that positive selection could be acting upon a subset of residues in these genes.

**Random Forest model**

RF is known to handle missing values; we performed additional data-curation steps to normalize the data as follows: each variant location that had identical values except in FH genomes were removed because these were identified as variants of reference genome FH and would not add any value in classification. Once the training dataset was divided in each fold, columns with all 0s or 1s were dropped as these monotonous columns cannot classify these genomes into separate clusters.

In a classification and regression tree algorithms such as Random Forest, the Gini impurity value is calculated when a randomly selected feature splits the dataset into two resulting in a correct classification. During model training, the calculated value reflects how often a feature is selected and decreases the overall impurity for each tree in the model. The Gini impurity decrease value can be averaged across all features, allowing features to be ranked by relative importance in correctly separating dataset into two sets at each node to minimize the entropy [9, 10]. The top features contribute the most in decreasing the average Gini impurity value; hence, such features hold higher importance in separation and distribution of data than others.

**References**

1. Gladman SS, Torsten. VelvetOptimiser : a multi-threaded Perl script for automatically optimising the three primary parameter options (K, -exp_cov, -cov_cutoff) for the Velvet de novo sequence assembler. 2012.

2. Langdon WB. Performance of genetic programming optimised Bowtie2 on genome comparison and analytic testing (GCAT) benchmarks. BioData Min. 2015;8(1):1. doi: 10.1186/s13040-014-0034-0. PubMed PMID: 25621011; PubMed Central PMCID: PMCPMC4304608.

3. Li H, Handsaker B, Wysoker A, Fennell T, Ruan J, Homer N, et al. The Sequence Alignment/Map format and SAMtools. Bioinformatics. 2009;25(16):2078-9. Epub 2009/06/10. doi: 10.1093/bioinformatics/btp352. PubMed PMID: 19505943; PubMed Central PMCID: PMCPMC2723002.

4. Winchell JM, Thurman KA, Mitchell SL, Thacker WL, Fields BS. Evaluation of three real-time PCR assays for detection of *Mycoplasma pneumoniae* in an outbreak investigation. J Clin Microbiol. 2008;46(9):3116-8.

5. Xiao L, Ptacek T, Osborne JD, Crabb DM, Simmons WL, Lefkowitz EJ, et al. Comparative genome analysis of *Mycoplasma pneumoniae*. BMC Genomics. 2015;16:610. doi: 10.1186/s12864-015-1801-0. PubMed PMID: 26275904; PubMed Central PMCID: PMC4537597.

6. Darling ACE, Mau B, Blattner FR, Perma NT. Mauve: Multiple Alignment of Conserved Genomic Sequence With Rearrangements. Genome Res. 2004;14(7):1394-403.

7. Darling AE, Mau B, Perna NT. progressiveMauve: multiple genome alignment with gene gain, loss and rearrangement. PLoS One. 2010;5(6):e11147. doi: 10.1371/journal.pone.0011147. PubMed PMID: 20593022; PubMed Central PMCID: PMCPMC2892488.

8. Lluch-Senar M, Cozzuto L, Cano J, Delgado J, Llorens-Rico V, Pereyre S, et al. Comparative "-omics" in *Mycoplasma pneumoniae* Clinical Isolates Reveals Key Virulence Factors. PLoS One. 2015;10(9):e0137354. doi: 10.1371/journal.pone.0137354. PubMed PMID: 26335586; PubMed Central PMCID: PMC4559472.

9. Dutilh BE, Thompson CC, Vicente AC, Marin MA, Lee C, Silva GG, et al. Comparative genomics of 274 *Vibrio cholerae* genomes reveals mobile functions structuring three niche dimensions. BMC Genomics. 2014;15:654. doi: 10.1186/1471-2164-15-654. PubMed PMID: 25096633; PubMed Central PMCID: PMCPMC4141962.

10. Menze BH, Kelm BM, Masuch R, Himmelreich U, Bachert P, Petrich W, et al. A comparison of random forest and its Gini importance with standard chemometric methods for the feature selection and classification of spectral data. BMC Bioinformatics. 2009;10:213. doi: 10.1186/1471-2105-10-213. PubMed PMID: 19591666; PubMed Central PMCID: PMCPMC2724423.
