# Supplementary material for: Correction: Comprehensive bioinformatics analysis of Mycoplasma pneumoniae genomes to investigate underlying population structure and type-specific determinants
Source: PLoS One. 2017 Oct 4;12(10):e0186030. doi: 10.1371/journal.pone.0186030 (PMC5627953; doi:10.1371/journal.pone.0186030)
Supplement: S2 Table — (DOCX) [file pone.0186030.s001.docx]

**S2 Table. NCBI BioProject, BioSample, SRA, and accession IDs for newly sequenced genomes and references used in current study.**

| **Sample ID** | **Sample Name** | **BioProjectID** | **BioSampleID** | **SRA Accession** | **NCBI Accession** |
| --- | --- | --- | --- | --- | --- |
| CO37 | CO37_USA_CO_2013 | PRJNA328823 | SAMN05391688 | SRR3924583 | N/A |
| OR1 | OR1_USA_OR_2011 | PRJNA328823 | SAMN05391689 | SRR3924584 | N/A |
| SA18 | SA18_SouthAfrica_2012 | PRJNA328823 | SAMN05391690 | SRR3924595 | N/A |
| FH 1965 | FH_USA_MA_1965 | PRJNA328823 | SAMN05391691 | SRR3924606 | MJJA00000000 |
| FH 2009 | FH_USA_MA_2009 | PRJNA328823 | SAMN05391692 | SRR3924617 | CP017327 |
| 685 | 685_Denmark_1988 | PRJNA328823 | SAMN05391693 | SRR3924628 | CP017328 |
| 988 | 988_Canada_1992 | PRJNA328823 | SAMN05391694 | SRR3924639 | N/A |
| E57 | E57_Egypt_2009 | PRJNA328823 | SAMN05391695 | SRR3924647 | CP017329 |
| G10 | G10_Guatemala_20110 | PRJNA328823 | SAMN05391696 | SRR3924648 | N/A |
| NM3 | NM3_USA_NM_2010 | PRJNA328823 | SAMN05391697 | SRR3924649 | N/A |
| 549 | 549_USA_WA_1965 | PRJNA328823 | SAMN05391698 | SRR3924585 | CP017330 |
| WV9 | WV9_USA_WV_2012 | PRJNA328823 | SAMN05391699 | SRR3924586 | N/A |
| FL8 | FL8_USA_FL_2012 | PRJNA328823 | SAMN05391700 | SRR3924587 | CP017331 |
| 986 | 986_Kenya_1998 | PRJNA328823 | SAMN05391701 | SRR3924588 | MJIZ00000000 |
| K21 | K21_Kenya_2010 | PRJNA328823 | SAMN05391702 | SRR3924589 | MJIY00000000 |
| G6 | G6_Guatemala_2010 | PRJNA328823 | SAMN05391703 | SRR3924590 | MJIX00000000 |
| E16 | E16_Egypt_2010 | PRJNA328823 | SAMN05391704 | SRR3924591 | CP017332 |
| 303 | 303_USA_AL_1991 | PRJNA328823 | SAMN05391705 | SRR3924592 | MJIW0000000 |
| FL1 | FL1_USA_FL_2012 | PRJNA328823 | SAMN05391706 | SRR3924593 | CP017333 |
| MA1 | MA1_USA_MA_2011 | PRJNA328823 | SAMN05391707 | SRR3924594 | N/A |
| K27 | K27_Kenya_2010 | PRJNA328823 | SAMN05391708 | SRR3924596 | CP017334 |
| CO103 | CO103_USA_CO_2013 | PRJNA328823 | SAMN05391709 | SRR3924597 | CP017335 |
| GA3 | GA3_USA_GA_2012 | PRJNA328823 | SAMN05391710 | SRR3924598 | CP017336 |
| 1005 | 1005_USA_NY_1999 | PRJNA328823 | SAMN05391711 | SRR3924599 | MJIV00000000 |
| 1006 | 1006_USA_NY_1999 | PRJNA328823 | SAMN05391712 | SRR3924600 | CP017337 |
| 987 | 987_USA_CA_1986 | PRJNA328823 | SAMN05391713 | SRR3924601 | N/A |
| 1134 | 1134_USA_IN_1999 | PRJNA328823 | SAMN05391714 | SRR3924602 | CP017338 |
| RI2 | RI2_USA_RI_2011 | PRJNA328823 | SAMN05391715 | SRR3924603 | N/A |
| 985 | 985_USA_SC_1988 | PRJNA328823 | SAMN05391716 | SRR3924604 | N/A |
| 519 | 519_USA_CA_1995 | PRJNA328823 | SAMN05391717 | SRR3924605 | CP017339 |
| 237 | 237_USA_OH_1993 | PRJNA328823 | SAMN05391718 | SRR3924607 | N/A |
| 682 | 682_Denmark_Unknown | PRJNA328823 | SAMN05391719 | SRR3924608 | MJIU00000000 |
| RI3 | RI3_USA_RI_2007 | PRJNA328823 | SAMN05391720 | SRR3924609 | CP017340 |
| 1801 | 1801_USA_DC_2000 | PRJNA328823 | SAMN05391721 | SRR3924610 | CP017341 |
| 334 | 334_USA_NJ_1994 | PRJNA328823 | SAMN05391722 | SRR3924611 | N/A |
| 3076 | 3076_USA_NH_2007 | PRJNA328823 | SAMN05391723 | SRR3924612 | N/A |
| 300 | 300_USA_NY_1994 | PRJNA328823 | SAMN05391724 | SRR3924613 | N/A |
| 709 | 709_USA_NY_1996 | PRJNA328823 | SAMN05391725 | SRR3924614 | N/A |
| O-360 | O360_USA_ME_2007 | PRJNA328823 | SAMN05391726 | SRR3924615 | N/A |
| 399 | 399_USA_PA_1994 | PRJNA328823 | SAMN05391727 | SRR3924616 | N/A |
| 2P | 2P_USA_RI_2007 | PRJNA328823 | SAMN05391728 | SRR3924618 | N/A |
| 386 | 386_USA_TX_1994 | PRJNA328823 | SAMN05391729 | SRR3924619 | N/A |
| 551 | 551_USA_WA_1974 | PRJNA328823 | SAMN05391730 | SRR3924620 | N/A |
| WI3 | WI3_USA_WI_2012 | PRJNA328823 | SAMN05391731 | SRR3924621 | N/A |
| WI6 | WI6_USA_WI_2012 | PRJNA328823 | SAMN05391732 | SRR3924622 | N/A |
| WV1 | WV1_USA_WV_2011 | PRJNA328823 | SAMN05391733 | SRR3924623 | N/A |
| NE4 | NE4_USA_NE_2014 | PRJNA328823 | SAMN05391734 | SRR3924624 | N/A |
| NE26 | NE26_USA_NE_2014 | PRJNA328823 | SAMN05391735 | SRR3924625 | N/A |
| NM1 | NM1_USA_NM_2010 | PRJNA328823 | SAMN05391736 | SRR3924626 | N/A |
| NM2 | NM2_USA_NM_2010 | PRJNA328823 | SAMN05391737 | SRR3924627 | N/A |
| CO13 | CO13_USA_CO_2013 | PRJNA328823 | SAMN05391738 | SRR3924629 | N/A |
| CO59 | CO59_USA_CO_2013 | PRJNA328823 | SAMN05391739 | SRR3924630 | N/A |
| CO36 | CO36_USA_CO_2013 | PRJNA328823 | SAMN05391740 | SRR3924631 | MJIT00000000 |
| CO3 | CO3_USA_CO_2014 | PRJNA328823 | SAMN05391741 | SRR3924632 | CP017342 |
| CO58 | CO58_USA_CO_2013 | PRJNA328823 | SAMN05391742 | SRR3924633 | N/A |
| CO26 | CO26_USA_CO_2013 | PRJNA328823 | SAMN05391743 | SRR3924634 | N/A |
| M129 | M129_USA_NC_1968 | PRJNA328823 | SAMN05391744 | SRR3924635 | CP017343 |
| EPC205 | EPC205_USA_UT_2012 | PRJNA328823 | SAMN05391745 | SRR3924636 | N/A |
| EPC83 | EPC83_USA_UT_2011 | PRJNA328823 | SAMN05391746 | SRR3924637 | N/A |
| EPC67 | EPC67_USA_TN_2012 | PRJNA328823 | SAMN05391747 | SRR3924638 | N/A |
| EPC181 | EPC181_USA_TN_2012 | PRJNA328823 | SAMN05391748 | SRR3924640 | N/A |
| EPC104 | EPC104_USA_UT_2012 | PRJNA328823 | SAMN05391749 | SRR3924641 | N/A |
| EPC37 | EPC37_USA_UT_2011 | PRJNA328823 | SAMN05391750 | SRR3924642 | N/A |
| EPC122 | EPC122_USA_IL_2012 | PRJNA328823 | SAMN05391751 | SRR3924643 | N/A |
| EPC164 | EPC164_USA_TN_2012 | PRJNA328823 | SAMN05391752 | SRR3924644 | N/A |
| EPC44 | EPC44_USA_UT_2011 | PRJNA328823 | SAMN05391753 | SRR3924645 | N/A |
| EPC230 | EPC230_USA_TN_2012 | PRJNA328823 | SAMN05391754 | SRR3924646 | N/A |
| FH^1^ | *Mycoplasma pneumoniae* FH | PRJNA224116 | SAMN03284384 | N/A | NZ_CP010546.1 |
| 309^1^ | DRS001148 | PRJNA224116 | SAMD00012611 | DRS001148 | NC_016807.1 |
| M129^1^ | U00089 | PRJNA57709 | SAMN02603304 | N/A | NC_000912.1 |
| M129-B7^1^ | CP003913 | PRJNA224116 | SAMN02604130 | N/A | NC_020076.2 |

^1^Reference genome
